# A BONCAT-iTRAQ method enables temporally resolved quantitative profiling of newly synthesised proteins in *Leishmania mexicana* parasites during starvation

**DOI:** 10.1371/journal.pntd.0007651

**Published:** 2019-12-19

**Authors:** Karunakaran Kalesh, Paul W. Denny

**Affiliations:** 1 Department of Chemistry, Durham University, Durham, United Kingdom; 2 Department of Biosciences, Durham University, Durham, United Kingdom; Bernhard Nocht Institute for Tropical Medicine, Hamburg, Germany, GERMANY

## Abstract

Adaptation to starvation is integral to the *Leishmania* life cycle. The parasite can survive prolonged periods of nutrient deprivation both *in vitro* and *in vivo*. The identification of parasite proteins synthesised during starvation is key to unravelling the underlying molecular mechanisms facilitating adaptation to these conditions. Additionally, as stress adaptation mechanisms in *Leishmania* are linked to virulence as well as infectivity, profiling of the complete repertoire of Newly Synthesised Proteins (NSPs) under starvation is important for drug target discovery. However, differential identification and quantitation of low abundance, starvation-specific NSPs from the larger background of the pre-existing parasite proteome has proven difficult, as this demands a highly selective and sensitive methodology. Herein we introduce an integrated chemical proteomics method in *L*. *mexicana* promastigotes that involves a powerful combination of the BONCAT technique and iTRAQ quantitative proteomics Mass Spectrometry (MS), which enabled temporally resolved quantitative profiling of *de novo* protein synthesis in the starving parasite. Uniquely, this approach integrates the high specificity of the BONCAT technique for the NSPs, with the high sensitivity and multiplexed quantitation capability of the iTRAQ proteomics MS. Proof-of-concept experiments identified over 250 starvation-responsive NSPs in the parasite. Our results show a starvation-specific increased relative abundance of several translation regulating and stress-responsive proteins in the parasite. GO analysis of the identified NSPs for Biological Process revealed translation (enrichment P value 2.47e^-35^) and peptide biosynthetic process (enrichment P value 4.84e^-35^) as extremely significantly enriched terms indicating the high specificity of the NSP towards regulation of protein synthesis. We believe that this approach will find widespread use in the study of the developmental stages of *Leishmania* species and in the broader field of protozoan biology.

## Author summary

Periodic nutrient scarcity plays crucial roles in the life cycle of the protozoan parasite *Leishmania spp*. Although adaptation to nutrient stress has a pivotal role in *Leishmania* biology, the underlying mechanisms remain poorly understood. In a period of nutrient starvation, the parasite responds by decreasing its protein production to conserve nutrient resources and to prevent formation of toxic proteins. However, even during severe starvation, the parasite generates certain essential quality control and rescue proteins. Differential identification of the complete repertoire of these proteins synthesised during starvation from the pre-existing proteins in the parasite holds the key to understanding the starvation adaptation mechanisms. This has been challenging to accomplish due to technical limitations. Using a combination of chemical labelling techniques and protein mass-spectrometry, we selectively identified and measured the proteins generated in the starving *Leishmania* parasite. Our results show a starvation time-dependent differential expression of important protein synthesis regulators in the parasite. This will serve as an important dataset for a holistic understanding of the starvation adaptation mechanisms in *Leishmania*. We also believe that this method will find widespread applications in the field of protozoa and other parasites causing Neglected Tropical Diseases.

## Introduction

Protozoan parasites of the *Leishmania spp*. are the causative agents of leishmaniasis, a Neglected Tropical Disease (NTD) endemic in over 90 countries worldwide, affecting approximately 12 million people with an estimated 700,000 to 1 million new cases annually [1]. These protozoa have a complex life cycle, progressing from extracellular promastigote stages in the sandfly vector to the obligate intramacrophage amastigote stage in the mammalian host [2]. During their digenetic life cycle *Leishmania spp*. are exposed to extreme stress conditions, including severe nutrient starvation, and the parasites have evolved mechanisms to adapt to and surmount large fluctuations in nutrient availability [3–5]. However, the key proteins involved in the starvation-adaptation mechanisms of the parasite remains unknown and the identification and quantitation of proteins synthesised *de novo* during starvation is critical to develop understanding of these. Progress in this direction has been hampered by technical limitations; the lack of availability of a robust and sensitive method that can differentiate and characterise the *de novo* synthesised proteins during starvation from the complex cellular background of pre-existing proteome being the major bottleneck. Herein we describe a combination of the bio-orthogonal non-canonical amino acid tagging (BONCAT) [6,7] technology and isobaric tags for relative and absolute quantification (iTRAQ) quantitative mass-spectrometry (MS) proteomics [8,9] to quantitatively profile the newly synthesised proteome (NSP) of *Leishmania mexicana* promastigotes during starvation.

Regulation of eukaryotic gene expression involves a coordinated network of molecular processes staring with initiation of transcription, followed by post-transcriptional regulatory mechanisms. Processing of the primary transcript-RNAs essentially involves three steps namely capping, where a 7-methylguanosine moiety modifies the 5’ end, and polyadenylation, where a poly-A tail is added at the 3’ end, and finally removal of the intron sequences via splicing. The processed RNAs (mRNAs) are then transported to the cytoplasm for the translation to take place. The transcripts interact with several proteins to form messenger ribonucleoprotein complexes (mRNPs), which regulate many aspects of mRNA stability and function. Intriguingly, regulation of gene expression in *Leishmania spp*., and other similar kinetoplastids, is fundamentally different from other eukaryotes [10–12]. As the open reading frames of genes in these parasites are arranged in long polycistronic clusters, RNA polymerase II-dependent regulation of transcription initiation does not occur and instead, monocistronic mRNAs are generated by a trans-splicing mechanism and polyadenylation. The gene expression in *Leishmania spp*. is regulated almost exclusively at the post-transcriptional level and this involves protein-mediated molecular mechanisms controlling the mRNA degradation, RNA editing in the kinetoplast and protein translation. Because of the existence of an independent layer of translational regulation, the mRNA levels in *Leishmania* are not a good predictor of protein expression, and this poor correlation between the transcript and protein expressions warrants in-depth protein-level studies in this organism [13,14].

The proteome of an organism is highly dynamic, and the protein turnover is tightly controlled by multiple check points in protein synthesis and degradation processes. This dynamic protein turnover plays a crucial role in maintaining the cellular homeostasis. Cells respond to stimuli and perturbations by altering their protein expression levels. Measuring these changes in the proteome is important to understanding the underlying biological processes. MS proteomics serves as a powerful technique for directly measuring the effect of perturbations on cellular proteome [15]. However, during starvation in *Leishmania*, the global protein synthesis operates at a lower rate as the parasite tries to conserve available limited nutrient resources, a highly robust and sensitive enrichment method has to be coupled with the MS in order to differentially identify the NSPs from the larger background of the parasite’s existing proteome.

We envisaged that the BONCAT technique could be applied for a selective profiling of NSPs in *L*. *mexicana* parasites during starvation. BONCAT involves metabolic incorporation of a methionine surrogate non-canonical amino acid bearing a small bio-orthogonal functional group, such as L-azidohomoalanine (AHA) or L-homopropargylglycine (HPG), into the newly synthesised proteins (NSPs). As AHA and/or HPG are methionine surrogates, they are readily and efficiently incorporated into NSPs by cell’s endogenous translational machinery [6]. In this case, the presence of the bio-orthogonal click tag in the newly translated proteins provide an efficient means to distinguish, and selectively isolate these proteins from the pre-existing pool of proteins via a highly efficient copper (I) catalysed azide-alkyne cycloaddition (CuAAC) click reaction [16] with a capture reagent bearing the orthogonal functionality to the tag (alkyne vs. azide and *vice versa*). Additionally, in order to get a temporally resolved quantitative information of the effect of starvation on the protein synthesis in the parasite, we decided to couple the BONCAT approach with iTRAQ quantitative proteomics MS [8]. The iTRAQ, similar to the tandem mass tag (TMT) quantitative proteomics [17], is a peptide-level labelling approach that offers sample multiplexing. Importantly, the multiplexed isobaric tags provide an advantage of pooling of peptide signals across the different test conditions, which increases the sensitivity of detection of even low-abundant peptides [18,19]. iTRAQ, and other similar labelling-based quantitative MS techniques, offer far more reliable and reproducible proteome quantitation than the different versions of spectral counting or precursor ion signal intensity-based label-free quantitative MS [20,21]. Using a combination of BONCAT and iTRAQ 4-plex quantitative MS, we have first identified and quantified a total of 166 NSPs in *L*. *mexicana* promastigotes under starvation. The iTRAQ 4-plex platform enabled profiling of relative quantitative changes in the abundance of the NSPs at three different time points after the onset of starvation in the parasite. Subsequent gene ontology (GO) analysis of the data sets revealed significant enrichment of proteins involved in regulating protein translation in the parasite. Using non-starved parasites as controls in a slightly larger-scale BONCAT-iTRAQ 3-plex experiments enabled further refinement of starvation-specific relative quantification of the parasite's NSPs and led to the identification of over 250 high confidence starvation-responsive NSPs in the parasite. This is the first quantitative proteomics study that revealed the NSPs along with their temporally resolved quantitative expression changes during starvation in *Leishmania*.

## Material and methods

### Chemicals and reagents

L-Azidohomoalanine (AHA; Iris Biotech GmbH), Cycloheximide (CHX; ACROS Organics), Tris (2-carboxyethy)phosphine hydrochloride (TCEP; Sigma Aldrich), 5-Tetramethylrhodamine-Alkyne (TAMRA-Alkyne; Jena Bioscience), Acetylene-PEG4-Biotin (Biotin-Alkyne; Jena Bioscience), Tris [(1-benzyl-1H-1,2,3-triazol-4-yl)methyl]amine (TBTA; Sigma Aldrich), Dimethyl sulfoxide (DMSO; Sigma Aldrich), Copper sulphate (CuSO_4_; Sigma Aldrich), 2-Amino-2-(hydroxymethyl)-1,3-propanediol (Tris; Sigma Aldrich), 4-(2-Hydroxyethyl)piperazine-1-ethanesulfonic acid (HEPES; Sigma Aldrich), Sodium chloride (NaCl; Fisher Scientific), Sodium dodecyl sulphate (SDS; Fisher Scientific), Sodium bicarbonate (NaHCO_3_; ACROS Organics), Calcium chloride (CaCl_2_; Sigma Aldrich), Urea (Sigma Aldrich), 1,4-Dithiothreitol (DTT; Sigma Aldrich), 2-Chloroacetamide (CAA; Sigma Aldrich), L-Glutamine solution (Sigma Aldrich), Benzonase (Sigma Aldrich), DC Protein Assay (Bio-Rad), Dialysed Foetal Bovine Serum (FBS; Life Technologies), Schneider’s Insect Medium (Sigma-Aldrich), Schneider’s Drosophila Medium without L-Methionine (PAN Biotech), Dulbecco Phosphate Buffered Saline (DPBS, Gibco), 1M Triethylammonium bicarbonate (TEAB) buffer (Sigma Aldrich), NeutrAvidin-Agarose beads (Thermo Scientific), iTRAQ Reagents Multiplex Kit (Sigma Aldrich), Optima LC-MS Grade Trifluoroacetic acid (TFA; Fisher Scientific), Optima LC-MS Grade Formic acid (FA; Fisher Scientific), Optima LC-MS Grade Acetonitrile (ACN; Fisher Scientific), Optima LC-MS Grade Methanol (MeOH; Fisher Scientific) and Sequencing Grade Modified Trypsin (Promega).

### Culturing of *L*. *mexicana* promastigotes

Promastigote form of *L*. *mexicana* strain M379 (MNYC/BC/62/M379) were grown in T-25 flasks at 26 ^o^C in Schneider’s Insect Medium (Sigma-Aldrich) supplemented with 0.4g/L NaHCO_3_, 0.6g/L anhydrous CaCl_2_ and 10% FBS (pH 7.2).

### Metabolic labelling of newly synthesised proteins in *L*. *mexicana* promastigotes

As AHA is a methionine surrogate, successful application of the BONCAT technique often requires depleting of L-methionine from the intracellular methionine reserves, and this is accomplished by maintaining the cells in a methionine-free medium for a short duration. For most applications, complete depletion of methionine from the cell is unnecessary as an excess concentration of the AHA that can outcompete with methionine for binding to the methionyl tRNA synthetase typically provide sufficient functional incorporation [22]. Therefore, BONCAT protocols typically employ only 20 to 30 minutes of pre-treatment of the cells in methionine-free medium and such a short duration of pre-treatment usually ensures robust metabolic incorporation of the methionine surrogate AHA into NSPs [23]. The promastigotes in T-25 flasks were grown to mid log phase (~5 x 10^6^ parasites/mL) and washed with methionine-free Schneider’s medium supplemented with 10% dialysed FBS. The parasites were then incubated with methionine-free Schneider’s medium supplemented with 10% dialysed FBS for 30 minutes to deplete the intracellular methionine reserves. The parasites were labelled with AHA (100μM and 1mM) in fresh methionine-free Schneider’s medium supplemented with 10% dialysed FBS for 1 hour with or without CHX (10μM). In order to induce starvation, the parasites, after the initial 30 minutes of methionine depletion, were incubated with DPBS for different duration (1 hour to 7 hours) and treated with AHA (50μM) in DPBS for 1 more hour. DMSO was used as a vehicle control for the AHA treatment. In order to probe the NSPs since the point of the onset of severe starvation, in one of the samples the 1 hour AHA incubation was carried out concurrently with the first 1 hour DPBS treatment. This condition is defined as the 1 hour starvation in the experiments. Following the AHA treatment, the parasites were lysed using lysis buffer (50mM HEPES, pH 7.4, 150mM NaCl, 4% SDS, 250U Benzonase) and the protein concentrations were determined using Bio-Rad DC Protein Assay.

### Click chemistry

Parasite lysates at 1mg/mL concentration were treated with freshly premixed click chemistry reaction cocktail [100μM capture reagent (TAMRA-Alkyne or Biotin-Alkyne (S1 Fig); 10mM stock solution in DMSO), 1mM CuSO_4_ solution (50mM stock solution in MilliQ water), 1mM TCEP solution (50mM stock solution in MilliQ water) and 100μM TBTA (10mM stock solution in DMSO)] for 3 hours at room temperature. Proteins were precipitated by adding methanol (4 volumes), chloroform (1.5 volumes) and water (3 volumes) and collected by centrifugation at 16,000 g for 5 minutes. The protein precipitates were washed twice with methanol (10 volumes; centrifugation at 16,000 g for 5 minutes to collect the pellets) and the supernatants were discarded. The protein pellets were air-dried at room temperature for 20 minutes and stored in -80 ^o^C freezer.

### In-gel fluorescence scanning

The air-dried protein pellets were suspended in resuspension buffer (4% SDS, 50mM HEPES pH 7.4, 150mM NaCl) to 1.33mg/mL final concentration. 4X Laemmli Sample Buffer (reducing) was added so that the final protein concentration was 1mg/mL. The samples were then boiled at 95 ^o^C for 8 minutes and allowed to cool to room temperature. The proteins were resolved by SDS-PAGE (12% SDS Tris-HCl gels; 20μg of protein was loaded per gel lane). The gels were scanned for fluorescence labelling using a GE typhoon 5400 gel imager.

### Affinity enrichment

The air-dried protein pellets obtained after click chemistry and protein precipitation were dissolved in phosphate buffered saline (PBS) with 2% SDS to 5mg/mL concentration by sonication. In a typical experiment, 300μg of the parasite lysate (or 400μg for the iTRAQ 3-plex experiments) after click chemistry and protein precipitation was resuspended in 50μL of the 2% SDS buffer. The samples were then diluted 20-fold with PBS so that the final SDS amount was 0.1%. The samples were centrifuged at 10,000 g for 5 minutes to remove insoluble debris and the clear soluble portion was used for the affinity enrichment. Typically, 30μL of NeutrAvidin-Agarose beads (40μL for the iTRAQ 3-plex experiments), freshly washed three times with 0.1% SDS buffer (0.1% SDS in PBS), were added to each of the sample and the mixtures were rotated on an end-over-end rotating shaker for 2 hours at room temperature. The beads were then washed 3 times with 1% SDS in PBS, 3 times with 6M urea in PBS, 3 times with PBS and once with 25mM TEAB buffer. Each washing was performed with 20 volumes of the washing solutions with respect to the bead volume and centrifugation of the beads between washing steps were carried out at 2,000 g for 1 minute at room temperature.

### On-bead reduction, alkylation and tryptic digestion

The beads after affinity enrichment were resuspended in 150μl of 25mM TEAB buffer and treated with 5mM TCEP (100mM stock solution in water) for 30 minutes at 50 ^o^C. The beads were washed once with 25mM TEAB buffer and resuspended in 150μl of 25mM TEAB buffer and treated with 10mM CAA (200mM stock solution in water) in dark for 20 minutes at room temperature. The beads were again washed with 25mM TEAB buffer and resuspended in 200μl of fresh 50mM TEAB buffer and treated with 5μg of sequencing grade modified trypsin at 37 ^o^C for 16 hours. The samples were centrifuged at 5,000 g for 5 minutes at room temperature to collect the supernatant. The beads were washed twice with 50% (v/v) ACN containing 0.1% (v/v) FA (50 μL for each wash) and mixed with the previous supernatant. The collected tryptic peptides were acidified to pH 3 using FA and evaporated to dryness. The peptides were then re-dissolved in 0.1% (v/v) FA solution in water and subjected to desalting on Pierce C-18 Spin Columns (Thermo Scientific; CN: 89873) following manufacturer’s instructions. The peptides were evaporated to complete dryness under a vacuum.

### iTRAQ labelling

The iTRAQ labelling of the dried and desalted tryptic peptides were carried out using the iTRAQ Reagents Multiplex Kit following manufacturer’s instructions with minor modifications. Briefly, the peptides were resuspended in equal amounts (30μL) of dissolution buffer (0.5M TEAB buffer supplied with the iTRAQ kit). 70μL of ethanol was added to each iTRAQ reagent vial pre-equilibrated to room-temperature. The contents of the iTRAQ reagents vials were then carefully and quickly transferred to the respective vials of peptide digests (iTRAQ 4-plex labelling: iTRAQ 114 to the DMSO control; iTRAQ 115 to 1 hour starvation; iTRAQ 116 to 2 hour starvation and iTRAQ 117 to 3 hour starvation. iTRAQ 3-plex labelling: iTRAQ 114 to no starvation control; iTRAQ 116 to 1 hour starvation and iTRAQ 117 to 2 hour starvation). The labelling reactions were performed for 1.5 hours at room-temperature following which, the reactions were quenched with 100mM Tris base (1M stock solution) for 15 minutes at room-temperature. The samples labelled with the different iTRAQ channels were then pooled into a fresh vial, and concentrated on speed-vac. The peptides were reconstituted in water with 0.1% (v/v) FA and 2% (v/v) ACN and subjected to desalting on C-18 Sep-Pak Classic cartridges (Waters; WAT051910) following manufacturer’s instructions. The eluted peptides were concentrated under vacuum and subjected to a second round of cleaning up on HILIC TopTip (PolyLC; TT200HIL) solid-phase extraction tips following manufacturer’s instructions. The eluted peptides were concentrated under vacuum and reconstituted in aqueous 0.1% (v/v) FA.

### LC-MS/MS analysis

The iTRAQ 4-plex labelled tryptic peptides were separated on an Eksigent nanoLC 425 operating with a nano-flow module using Waters nanoEase HSS C18 T3 column (75μm x 250mm). A Waters trap column (Acquity M-Class Symmetry C18 5μm, 180μm x 20mm) was used prior to the main separating nano column. 2.5μL of sample peptides were separated by mobile phase A (0.1% formic acid in water) and mobile phase B (0.1% formic acid in ACN) at a flow rate of 300nL/minute over 110 minutes. The gradient used was the following, 5% B to 8% B (0 to 2 minutes), 8% B to 30% B (2 to 60 minutes), 30% B to 40% B (60 to 70 minutes), 40% B to 85% B (70 to 72 minutes), at 85% (72 to 78 minutes), 85% B to 5% B (78 to 80 minutes), at 5% B (80 to 110 minutes). The MS analysis was performed on a TripleTOF 5600 system (Sciex) in high-resolution mode. The MS acquisition time was set from gradient time 0 to 90 minutes and the MS spectra were collected in the mass range of 400 to 1600 m/z with 250ms accumulation time per spectrum. Further fragmentation of each MS spectrum occurred with a maximum of 30 precursors per cycle and 33ms minimum accumulation time for each precursor across the range of 100 to 1600 m/z and dynamic exclusion for 12sec. The MS/MS spectra were acquired in high sensitivity mode and the collision energies were increased by checking the ‘adjust CE when using iTRAQ reagents’ box in the acquisition method.

The iTRAQ 3-plex labelled tryptic peptides were separated on an ekspert nanoLC 425 with low micro gradient flow module (Eksigent) using a YMC-Triart C18 column (12nm, S-3μm, 150 x 0.3mm ID, 1/32"; Part number: TA12S03-15H0RU). A C-18 trap column (Trap-YMC-Triart 12nm S-5μm, 5 x 0.5mm ID, 1/32"; Part number: TA12S05-E5J0RU) was used prior to the main separating micro-flow column. 5μL of sample peptides were separated by mobile phase A (0.1% formic acid in water) and mobile phase B (0.1% formic acid in ACN) at a flow rate of 5μL/minute over 87 minutes. The gradient used was the following, 3% B to 5% B (0 to 2 minutes), 5% B to 30% B (2 to 68 minutes), 30% B to 35% B (68 to 73 minutes), 35% B to 80% B (73 to 75 minutes), at 80% (75 to 78 minutes), 80% B to 3% B (78 to 79 minutes), at 3% B (79 to 87 minutes). The MS analysis was performed on a TripleTOF 5600 system (Sciex) in high-resolution mode. The MS acquisition time was set from gradient time 0 to 85 minutes and the MS1 spectra were collected in the mass range of 400 to 1600 m/z with 250ms accumulation time per spectrum. Further fragmentation of each MS spectrum occurred with a maximum of 30 precursors per cycle and 33ms minimum accumulation time for each precursor across the range of 100 to 1500 m/z with ion selection +2 to +5, 500 cps intensity threshold and dynamic exclusion for 15sec. The MS/MS spectra were acquired in high sensitivity mode as described above.

### iTRAQ quantitative proteomics MS data processing and analysis

For protein identification and quantification, the wiff files from the Sciex TripleTOF 5600 system were imported into MaxQuant [24] (version 1.6.3.4) with integrated Andromeda database search engine [25]. The MS/MS spectra were queried against *L*. *mexicana* sequences from UniProt KB (8,524 sequences). Database search employed the following parameters: Reporter ion MS2 with multiplicity 4plex for the iTRAQ 4-plex experiments and multiplicity 3plex for iTRAQ 3-plex experiments, trypsin digestion with maximum 2 missed cleavages, oxidation of methionine and acetylation of protein N-termini as variable modifications, carbamidomethylation of cysteine as fixed modification, maximum number of modifications per peptide set at 5, minimum peptide length of 6, and protein FDR 0.01. Appropriate correction factors for the individual iTRAQ channels for both peptide N-terminal labelling and lysine side-chain labelling as per the iTRAQ Reagent Multiplex Kit were also configured into the database search. The proteinGroups.txt file from the MaxQuant search output was processed using Perseus software [26] (version 1.6.2.3). Potential contaminants, reverse sequences and sequences only identified by site were filtered off. A One-sample t-test was performed on the two replicates and only proteins with p ≤ 0.05 were retained. Additionally, only proteins with at least 2 unique peptides identified were retained. For each identified protein, ratios of the AHA labelled Reporter Intensity Corrected vs. DMSO control sample from the corresponding replicate experiment was calculated yielding the fold change (FC). The FCs obtained for each protein were transformed into log2 scale, and volcano plots were generated between the calculated significance (-Log P-value) and the obtained FC in log2 scale for each protein across the different durations of starvation.

### Gene Ontology analysis

The GO terms (Molecular Function, Biological Process, and Cellular Component) significantly enriched in the NSPs relative to the predicted *L*. *mexicana* proteome were derived using Trytripdb.org [27]. REVIGO software [28] (revigo.irb.hr) was employed to refine and visualise the enriched GO terms.

## Results

### AHA is metabolically incorporated into NSPs in *L*. *mexicana* promastigotes

**Fig 1:**
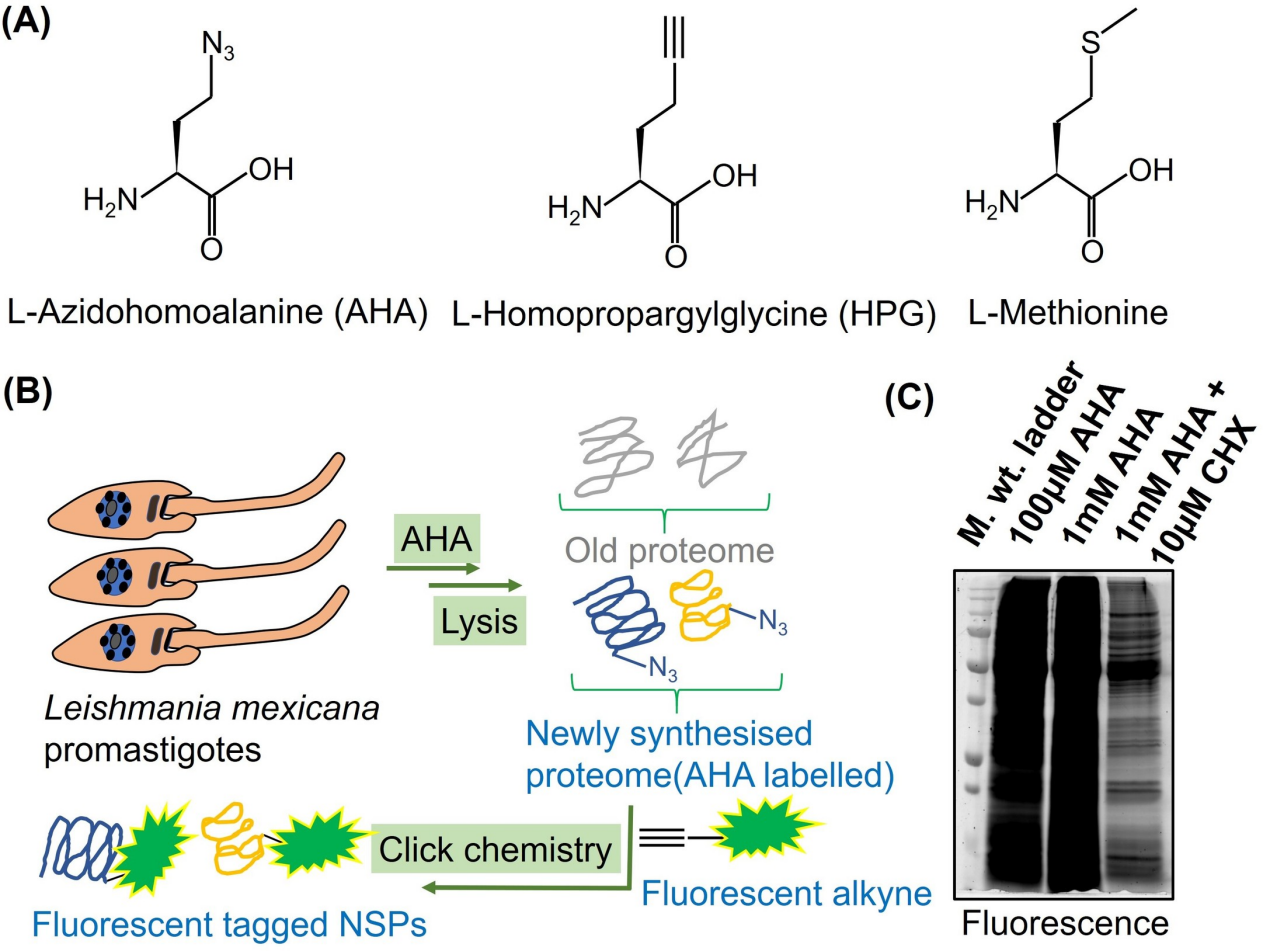
BONCAT in *L*. *mexicana* promastigotes. (**A**) Chemical structure of AHA, HPG and Methionine. (**B**) Workflow for BONCAT in *L*. *mexicana* promastigotes. AHA that can be bioorthogonally tagged with a fluorescent terminal alkyne was used for the BONCAT. (**C**) Fluorescent labelling of the NSPs following BONCAT detected by in-gel fluorescence scanning.

Although the BONCAT approach has been extensively applied in mammalian cells, reports are relatively few in lower eukaryotes. Therefore, we first decided to test if AHA (Fig 1A) is metabolically incorporated into NSPs in *L*. *mexicana* promastigotes. A general workflow for the metabolic incorporation of AHA into NSPs of *L*. *mexicana* promastigotes is presented (Fig 1B).The AHA incorporated NSPs preferentially tagged via biorthogonal click reaction with a TAMRA fluorophore-alkyne were visualised by in-gel fluorescence scanning. As shown in Fig 1C, intense fluorescence labelling of the NSP was observed even at the lower concentration of 100μM AHA treatment. Labelling saturation was observed at the higher concentration of 1mM AHA treatment. Importantly, even for the high concentration of 1mM AHA treatment, concurrent treatment of the protein synthesis inhibitor CHX significantly diminished the protein labelling, indicating that the fluorescently labelled proteins are indeed newly synthesised.

### Metabolic incorporation of AHA into the NSP of *L*. *mexicana* promastigotes is sensitive to starvation

**Fig 2:**
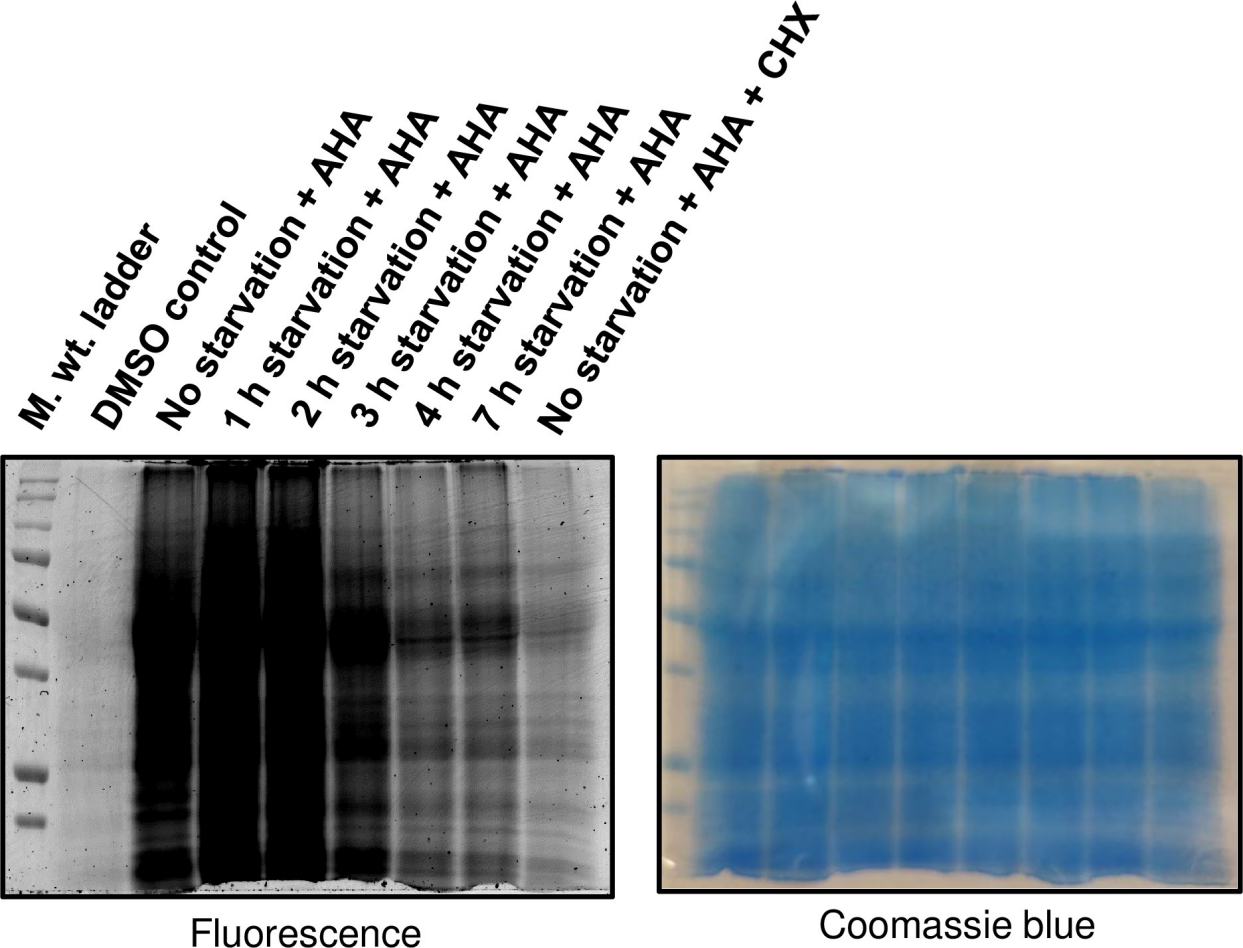
BONCAT in *L*. *mexicana* promastigotes under starvation. Promastigotes were cultured in methionine-free Schneider’s medium (30 minutes) prior to incubation in DPBS for different time periods (1 hour to 7 hours). The starved parasites were treated with AHA (50μM; lanes 3 to 9) or DMSO (control; lane 2) with (lane 9) or without CHX (10μM) for the last 1 hour of starvation and the NSPs were profiled by in-gel fluorescence scanning following click chemistry with a TAMRA-Alkyne. A Coomassie blue staining of the same gel that demonstrates even loading across the gel lanes is shown on the right panel.

In order to test whether the AHA incorporation can be used for the labelling of NSPs during starvation in *L*. *mexicana* promastigotes, we incubated the parasites in DPBS for different time durations prior to the AHA treatment. Maintaining the parasites in DBPS without serum ensures severe starvation [29]. As shown in Fig 2, starvation-time-dependent decrease in the fluorescent labelling intensity was observed in the in-gel fluorescence scans, indicating that the AHA incorporation can be used for the labelling of the NSPs under starvation in this parasite.

### Development of a BONCAT-iTRAQ 4-plex workflow for quantitative proteomics MS profiling of the NSP during starvation in *L*. *mexicana* promastigotes

**Fig 3:**
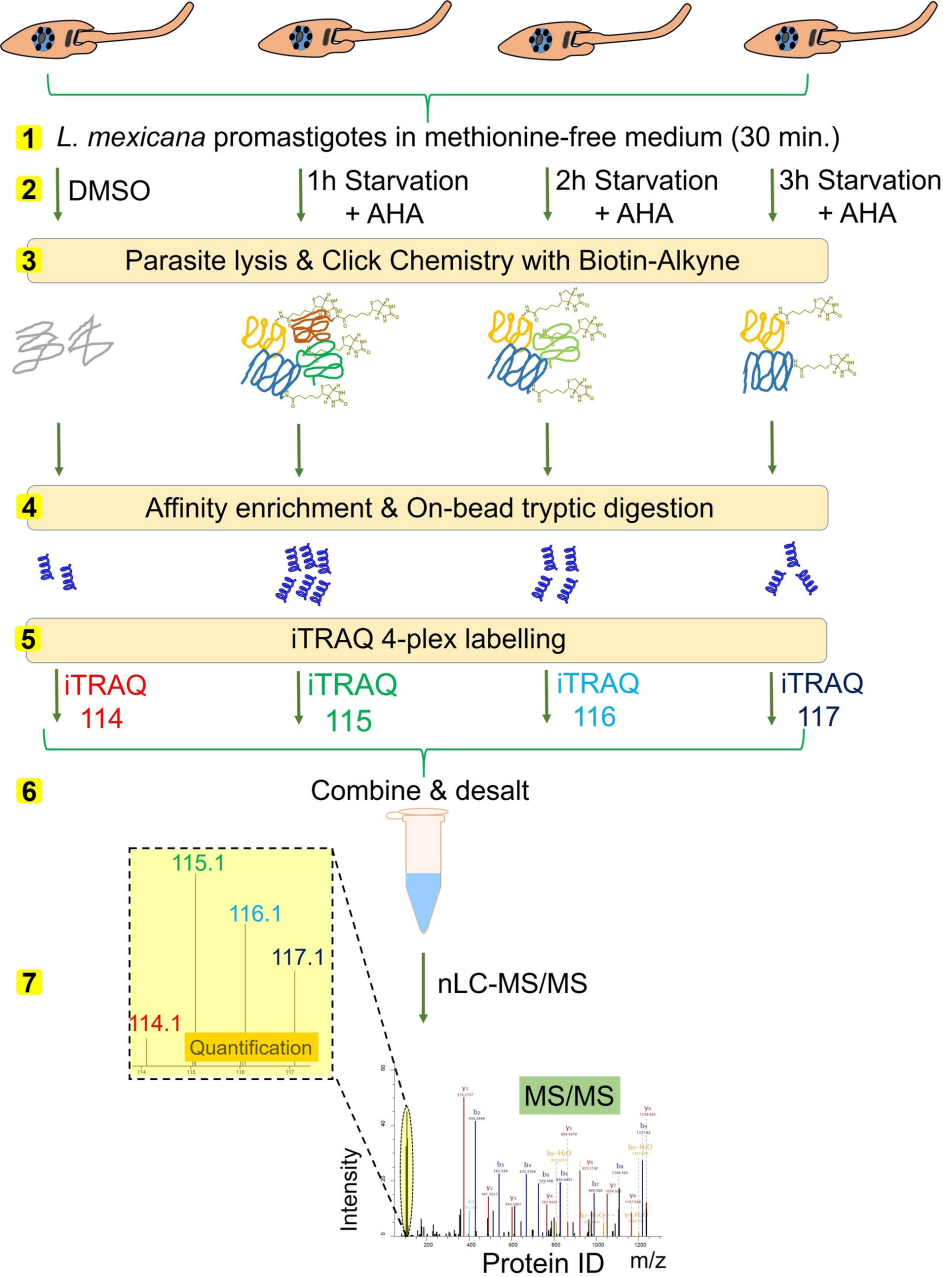
Schematic representation of the integrated BONCAT-iTRAQ 4-plex workflow. Parasites maintained in methionine-free medium for 30 minutes (step 1) were subjected to starvation in DPBS for 1, 2 and 3 hour durations, and the NSPs in the parasites were metabolically labelled with AHA treatment or DMSO treatment as control (step 2). The parasites were then lysed, and the NSPs were chemically tagged using bio-orthogonal click reaction with a Biotin-Alkyne (step 3). The biotin-tagged proteins were affinity enriched on NeutrAvidin beads, and following on-bead tryptic digestion (step 4), the released peptides were subjected to iTRAQ 4-plex labelling (step 5). iTRAQ channel 114 was used for labelling the DMSO control sample, whilst channels 115, 116 and 117 were used respectively for labelling the NSPs at 1 hour, 2 hour and 3 hour starvation. The samples after pooling together (step 6) were analysed by nanoLC-MS/MS (step 7). The relative intensity values of the reporter ions (iTRAQ ions 114, 115, 116 and 117) in the tandem mass spectra (MS/MS) of each detected tryptic peptide provide estimation of the relative abundance of the peptide in the corresponding sample.

The in-gel fluorescence scanning only provides a qualitative information of the differential AHA labelling under starvation. In order to identify and generate a comparative quantitation of the NSPs at different time-points of starvation, we coupled the iTRAQ quantitative proteomics MS with the BONCAT. We used iTRAQ 4-plex labelling that enables comparison of 4 different experimental conditions in one experiment. As starvation beyond 3 hour duration was found to generate very little protein labelling in this parasite (Fig 2), we decided to compare the 1 hour, 2 hour and 3 hour time periods of starvation using quantitative proteomics. As shown in Fig 3, *L*. *mexicana* promastigotes, following the three different durations of incubation with DPBS, were treated with AHA to label the NSP. DMSO treatment instead of AHA was used as a control. The parasite lysates were subjected to click chemistry with a Biotin-Alkyne capture reagent (S1 Fig) and the labelled proteome were affinity enriched on NeutrAvidin-Agarose beads. The strong non-covalent interaction between biotin in the capture reagent and NeutrAvidin (K_d_ ≈ 10^-15^M) [30] permits stringent washing steps during the affinity enrichment protocol, enabling highly robust and selective pull-down of the labelled NSPs. After on-bead reduction, alkylation and tryptic digestion, the peptide digests were subjected to labelling with iTRAQ 4-plex reagents. The samples were then combined, desalted and analysed by nanoLC-MS/MS.

### Identification and time-resolved quantitation of NSPs in *L*. *mexicana* promastigotes during starvation

**Fig 4:**
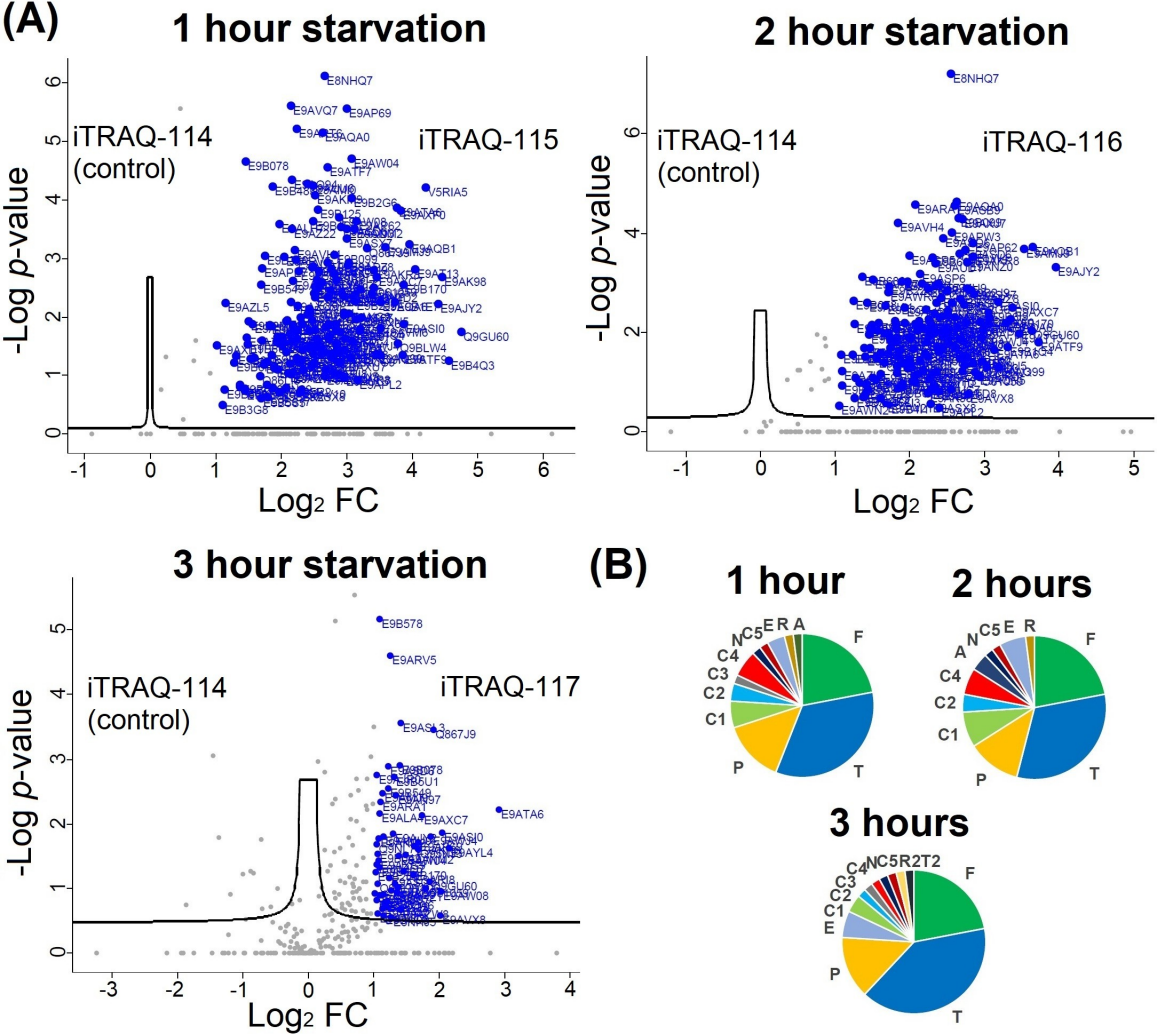
iTRAQ 4-plex quantitative proteomics MS profiling of NSPs of *L*. *mexicana* promastigotes during starvation. (**A**) Volcano plots of the NSPs detected at the three durations of starvation. The significance of the iTRAQ reporter intensities obtained for each NSP at each tested duration of starvation across two replicates as -Log P-values was plotted against the observed fold change (FC) in abundance in log2 scale. Proteins with a log2 FC of more than 1 with significant iTRAQ quantifications are highlighted in blue. (**B**) Functional annotation pie chart of the top-50 NSPs. The letter codes used for the functional categories are the following. (T) Translation, ribosomal structure and biogenesis; (F) Function unknown; (P) Post-translational modification, protein turnover, and chaperones; (A) Amino acid transport and metabolism; (C1) Carbohydrate transport and metabolism; (C2) Coenzyme transport and metabolism; (C3) Chromatin structure and dynamics; (C4) Cytoskeleton; (C5) Cell wall/membrane/envelope biogenesis; (R) Replication, recombination and repair; (R2) RNA processing and modification; (N) Nucleotide transport and metabolism; (T2) Transcription; (E) Energy production and conversion.

As shown in S1 Table, over 300 proteins were identified across the two replicate BONCAT-iTRAQ 4-plex experiments, of which 166 protein quantifications were statistically significant in a t-test analysis. For each of these NSPs, the iTRAQ reporter intensity ratio at each tested starvation duration to the DMSO control iTRAQ reporter intensity (iTRAQ 114 channel) within the same experiment was calculated. The observed fold change (FC) in abundance of each protein, after converting to log_2_ scale, was plotted against the significance in the t-test (-Log P-value). This enables filtering of the NSPs most significantly influenced by each tested duration of starvation (highlighted in blue in Fig 4A). A global decrease in the *de novo* protein synthesis was observed with increase in the duration of starvation. Functional annotation of the top-50 proteins by eggNOG database [31] revealed “translation, ribosomal structure and biogenesis” and “posttranslational modification, protein turnover and chaperons” along with “protein function unknown” as the most abundant classifications as depicted in the pie chart Fig 4B. The top-50 proteins with the highest changes in their abundance at each of the three tested duration of starvation are listed in the S2 Table. As shown in S2 Table, many translation regulating proteins were observed among the top-ranking proteins at all three tested durations of starvation. Our data also show that at different durations of starvation in the parasite, a panel of important translation regulator proteins are expressed to different abundance levels.

### A focussed BONCAT-iTRAQ 3-plex workflow enables identification of starvation-responsive NSPs

**Fig 5:**
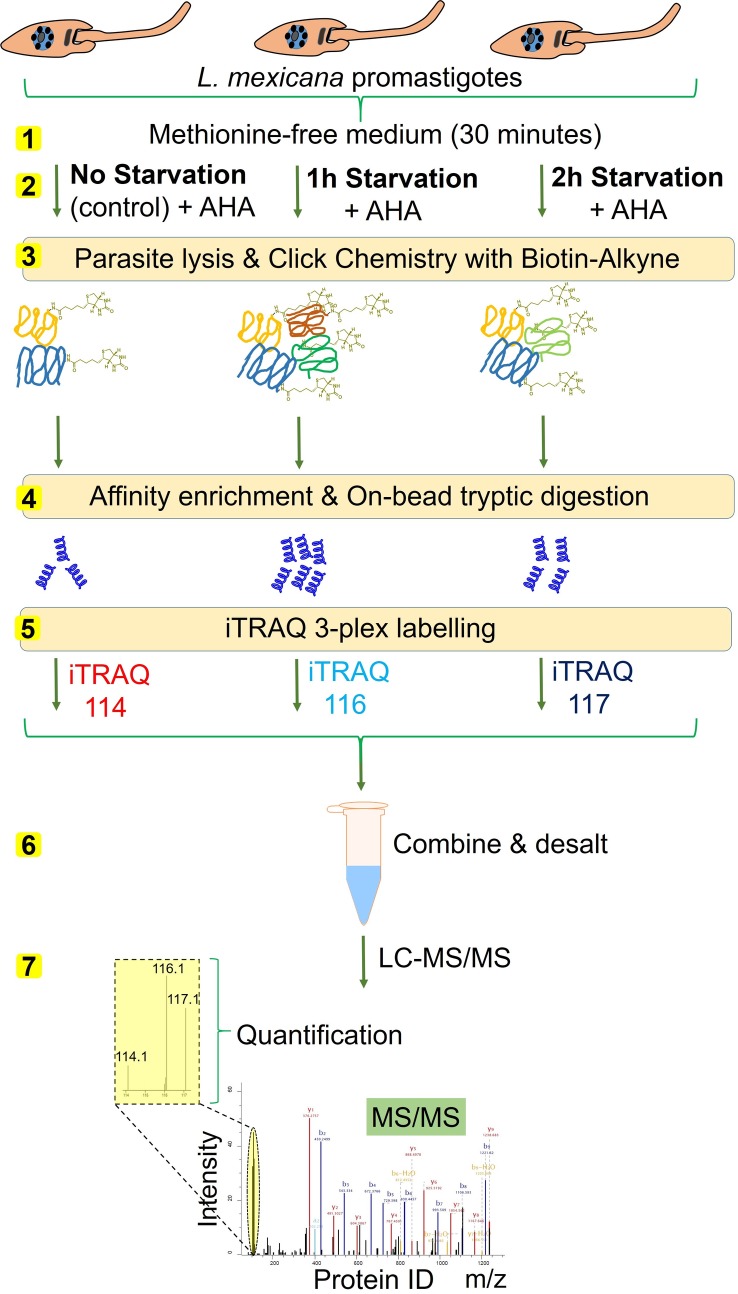
Schematic representation of BONCAT-iTRAQ 3-plex workflow for the identification of starvation-responsive NSPs. Parasites maintained in methionine-free medium for 30 minutes (step 1) were subjected to starvation in DPBS for 1 hour and 2 hour durations and the NSPs in the parasites were metabolically labelled with AHA treatment (step 2). Parallel AHA treatment on non-starved parasites were performed as controls. The parasites were then lysed, and the NSPs from the starved and non-starved batches were chemically tagged using click reaction with Biotin-Alkyne (step 3). The biotin-tagged proteins were affinity enriched on NeutrAvidin beads and following on-bead tryptic digestion (step 4), the released peptides were subjected to iTRAQ 3-plex labelling (step 5). iTRAQ channel 114 was used for labelling the AHA-treated non-starved control sample, whilst channels 116, and 117 were used respectively for labelling the NSPs at 1 hour and 2 hour durations of starvation. The samples after pooling together (step 6) were analysed by LC-MS/MS (step 7).

**Fig 6:**
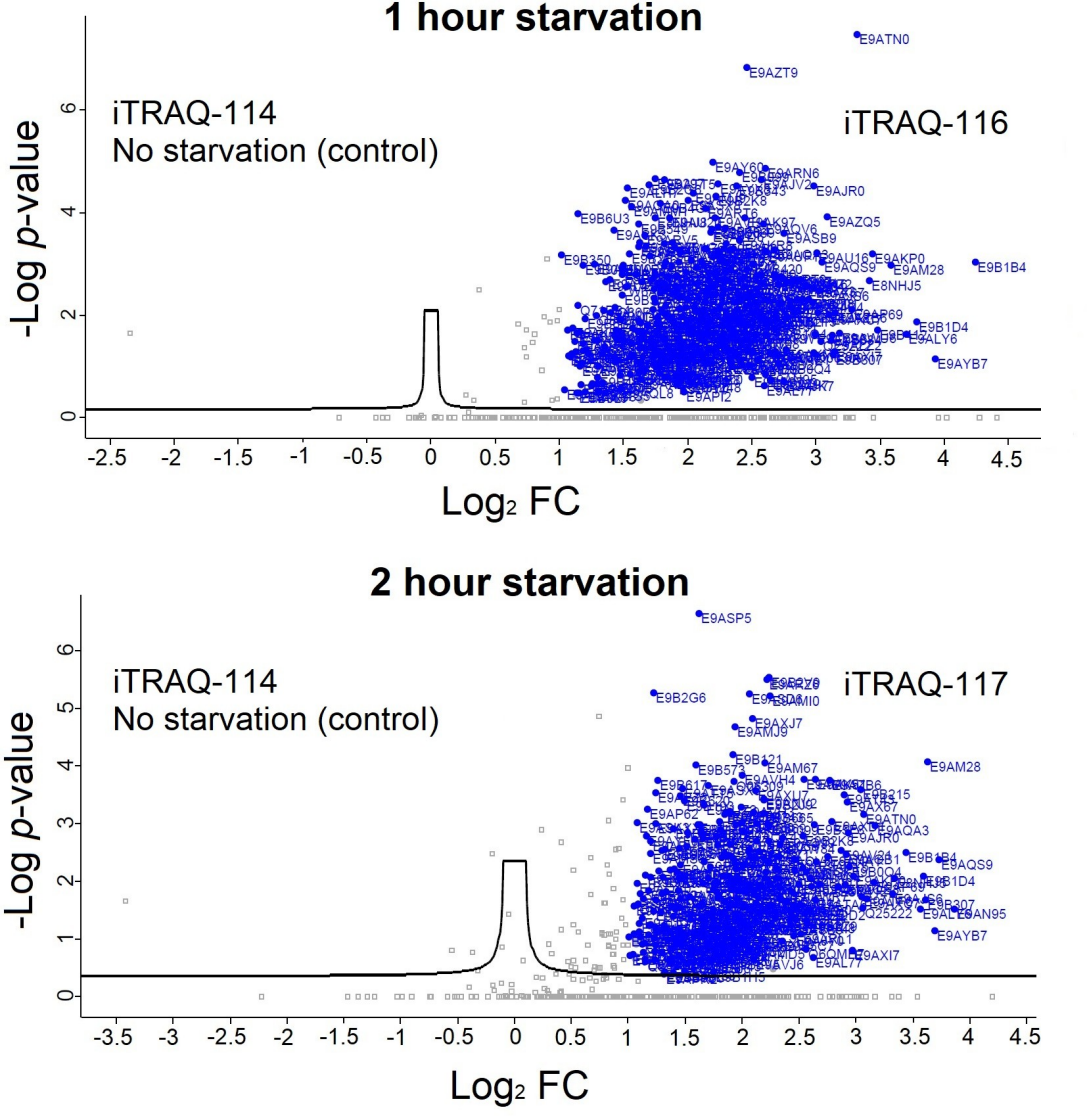
Starvation-responsive NSPs of *L*. *mexicana* promastigotes. Volcano plots of the starvation-responsive NSPs detected at 1 hour and 2 hour durations of starvation. The significance of the iTRAQ reporter intensities obtained for each NSP at 1 hour and 2 hours of starvation across two replicates as -Log P-values was plotted against the observed fold change (FC) in abundance in log_2_ scale. Proteins with a log_2_ FC of more than 1 with significant iTRAQ quantifications are highlighted in blue.

In order to further refine the identification of starvation-responsive NSPs in the parasite, we developed a BONCAT-iTRAQ 3-plex workflow (Fig 5) in which the DMSO vehicle treatment used in the previous experiments was replaced with AHA treated non-starved parasites as controls. This enabled direct relative iTRAQ quantitation of the NSPs at 1 hour and 2 hour durations of starvation against the NSPs of the non-starved parasites. As the global protein synthesis in the parasite was observed to be severely downregulated beyond the 2 hour period of nutrient deprivation (S1 Table, Fig 4), the maximum starvation window was restricted to 2 hours to minimise the missing values in the iTRAQ quantitation, enabling more robust and reliable relative quantitation. As shown in S3 Table, over 650 proteins were identified with at least 3 minimum valid values of the reporter intensities across the two replicate BONCAT-iTRAQ 3-plex experiments. After filtering for t-test significance (P ≤ 0.05) and minimum 2 unique peptides, a total of 356 and 299 proteins were respectively retained for the 1 hour and 2 hour starvation windows. Statistically significant NSPs with significant FC in abundance of >1 (in log_2_ scale) relative to the non-starved parasite controls are highlighted in blue filled circles in Fig 6. The complete list of proteins and their observed FC in abundance values are provided in S3 Table. We then functionally classified the NSPs (S4 Table, S2 Fig) and the major functional groups of important NSPs along with their observed FC in abundances are listed in Table 1.

**Table 1:** List of starvation-responsive NSPs by selected functional groups^a^.

Category and Gene ID	Protein name	log_2_ FC, 2h	log_2_ FC, 1h
**Protein folding/chaperones**			
LmxM.26.1380	Prefoldin subunit 3	3.695	3.924
LmxM.31.2260	Heat shock protein Hsp20, putative	3.610	3.131
LmxM.06.0120	Peptidyl-prolyl cis-trans isomerase	2.976	3.440
LmxM.28.1200	Putative glucose-regulated protein 78	2.463	2.459
LmxM.28.2770	Putative heat-shock protein hsp70	2.234	2.405
LmxM.31.3270	Putative chaperonin alpha subunit	2.068	2.301
LmxM.32.2390	Putative heat shock protein	2.057	2.221
LmxM.34.3860	T-complex protein 1 subunit eta	1.949	2.232
LmxM.18.1370	Putative heat shock protein	1.944	2.254
LmxM.29.2490	Putative heat shock 70-related protein 1, mitochondrial	1.915	2.183
LmxM.36.6910	Putative T-complex protein 1, theta subunit	1.899	1.993
LmxM.23.1220	T-complex protein 1 subunit gamma	1.783	2.110
LmxM.36.2030	Chaperonin HSP60, mitochondrial	1.702	2.003
LmxM.10.0890	Peptidylprolyl isomerase	1.643	1.777
LmxM.36.2020	Chaperonin HSP60, mitochondrial	1.573	1.653
LmxM.31.1000	Chaperonin containing t-complex protein,putative	1.552	2.119
LmxM.13.1660	Putative chaperonin TCP20	1.452	1.757
LmxM.29.0730	GrpE protein homolog	1.323	1.250
LmxM.26.1240	Heat shock protein 70-related protein	1.290	1.771
LmxM.27.1260	Putative T-complex protein 1, beta subunit	1.290	1.908
LmxM.32.0312	Heat shock protein 83–1	1.173	1.931
**Energy production**			
LmxM.33.3670	Vacuolar ATP synthase catalytic subunit A,putative	2.630	2.471
LmxM.18.0510	Aconitate hydratase	2.571	2.280
LmxM.34.1180	Putative NADH-dependent fumarate reductase	2.472	2.620
LmxM.24.0761	Malic enzyme	2.437	2.344
LmxM.28.1140	Putative electron-transfer-flavoprotein, alpha polypeptide	2.289	2.524
LmxM.36.5910	2,3-diketo-5-methylthio-1-phosphopentane phosphatase, putative	2.248	2.332
LmxM.25.2140	Succinate—CoA ligase [ADP-forming] subunit alpha, mitochondrial	2.122	2.302
LmxM.25.1180	ATP synthase subunit beta	2.087	2.305
LmxM.05.0350	Trypanothione reductase	2.062	2.703
LmxM.27.0880	Putative 2-oxoglutarate dehydrogenase subunit	2.005	2.071
LmxM.21.1770	Putative ATP synthase F1 subunit gamma protein	1.993	2.314
LmxM.36.3100	Putative ATP synthase	1.950	2.473
LmxM.36.2950	Succinate—CoA ligase [ADP-forming] subunit beta, mitochondrial	1.945	2.287
LmxM.29.2490	Malate dehydrogenase	1.923	2.334
LmxM.21.0550	Dihydrolipoamide acetyltransferaselike protein	1.896	2.085
LmxM.31.3310	Dihydrolipoyl dehydrogenase	1.893	1.830
LmxM.25.1120	Aldehyde dehydrogenase, mitochondrial	1.852	2.488
LmxM.12.1130	NADH:flavin oxidoreductase/NADH oxidase,putative	1.799	1.901
LmxM.24.1630	Succinate dehydrogenase [ubiquinone] flavoprotein subunit, mitochondrial	1.778	1.966
LmxM.28.2420	Dihydrolipoamide acetyltransferase component of pyruvate dehydrogenase complex	1.651	2.308
LmxM.32.2550	Isocitrate dehydrogenase [NADP]	1.648	1.430
LmxM.05.0500	Putative ATPase alpha subunit	1.631	2.254
LmxM.30.1220	Putative vacuolar-type proton translocating pyrophosphatase 1	1.607	1.916
LmxM.18.0670	Citrate synthase	1.447	1.339
LmxM.28.2430	Putative vacuolar ATP synthase subunit b	1.416	2.081
LmxM.27.1810	Glycosomal phosphoenolpyruvate carboxykinase,putative	1.283	1.466
**Translation**			
LmxM.07.0640	Eukaryotic translation initiation factor 3 subunit h	2.628	2.599
LmxM.31.2180	Eukaryotic translation initiation factor 3 subunit k	2.566	2.646
LmxM.09.1070	Putative eukaryotic translation initiation factor 2 subunit	2.542	2.707
LmxM.18.0740	Putative elongation factor Tu	2.514	1.821
LmxM.17.1290	Putative translation initiation factor	2.444	2.372
LmxM.12.0250	Putative cysteinyl-tRNA synthetase	2.187	1.940
LmxM.03.0980	Elongation initiation factor 2 alpha subunit,putative	2.130	2.416
LmxM.36.0180	Elongation factor 2	2.068	2.286
LmxM.08.0550	Translation initiation factor-like protein	2.066	2.422
LmxM.17.0010	Eukaryotic translation initiation factor 3 subunit a	1.898	2.330
LmxM.31.0870	Phenylalanyl-tRNA synthetase alpha chain,putative	1.894	2.016
LmxM.36.5620	Putative isoleucyl-tRNA synthetase	1.825	2.102
LmxM.27.1310	Putative arginyl-tRNA synthetase	1.820	2.274
LmxM.15.0230	Lysine—tRNA ligase	1.764	1.647
LmxM.18.1210	Putative prolyl-tRNA synthetase	1.762	1.650
LmxM.36.0250	Eukaryotic translation initiation factor 3 subunit l	1.659	1.841
LmxM.36.3840	Putative glycyl tRNA synthetase	1.549	1.955
LmxM.10.1080	Eukaryotic translation initiation factor 4 gamma 5	1.517	1.994
LmxM.11.0100	Putative seryl-tRNA synthetase	1.460	2.068
LmxM.33.2340	Putative asparaginyl-tRNA synthetase	1.457	2.171
LmxM.34.1410	Putative threonyl-tRNA synthetase	1.355	1.185
LmxM.19.0160	Putative aminopeptidase	1.328	1.899
LmxM.36.6980	Eukaryotic translation initiation factor 3 subunit c	1.234	1.753
LmxM.29.3130	Putative valyl-tRNA synthetase	1.177	1.301
**Signal transduction**			
LmxM.10.0200	Putative mitogen-activated protein kinase	2.591	2.549
LmxM.11.0350	Putative 14-3-3 protein	2.152	2.186
LmxM.34.1010	Putative casein kinase	2.149	2.329
LmxM.36.3180	Receptor-type adenylate cyclase a-like protein	2.034	2.329
LmxM.21.1080	Cell division protein kinase 2 homolog CRK1	1.928	2.984
LmxM.25.0750	Protein phosphatase, putative	1.696	2.003
LmxM.33.2820	Regulatory subunit of protein kinase a-like protein	1.590	1.472
**Vesicular transport**			
LmxM.10.0850	Putative nuclear transport factor 2	3.864	2.601
LmxM.16.1180	Coatomer subunit delta	3.729	3.046
LmxM.08_29.0880	Putative ADP ribosylation factor 3	3.563	3.702
LmxM.05.0030	Putative small GTP-binding protein	1.467	1.854

^a^Selected functional groups of the starvation-responsive NSPs at 1 hour and 2 hour durations of starvations are listed along with their observed fold change (FC) in abundance (log_2_ scale) relative to protein expression in non-starved parasites.

### Gene Ontology (GO) analysis of starvation-responsive NSPs in *L*. *mexicana* promastigotes

**Fig 7:**
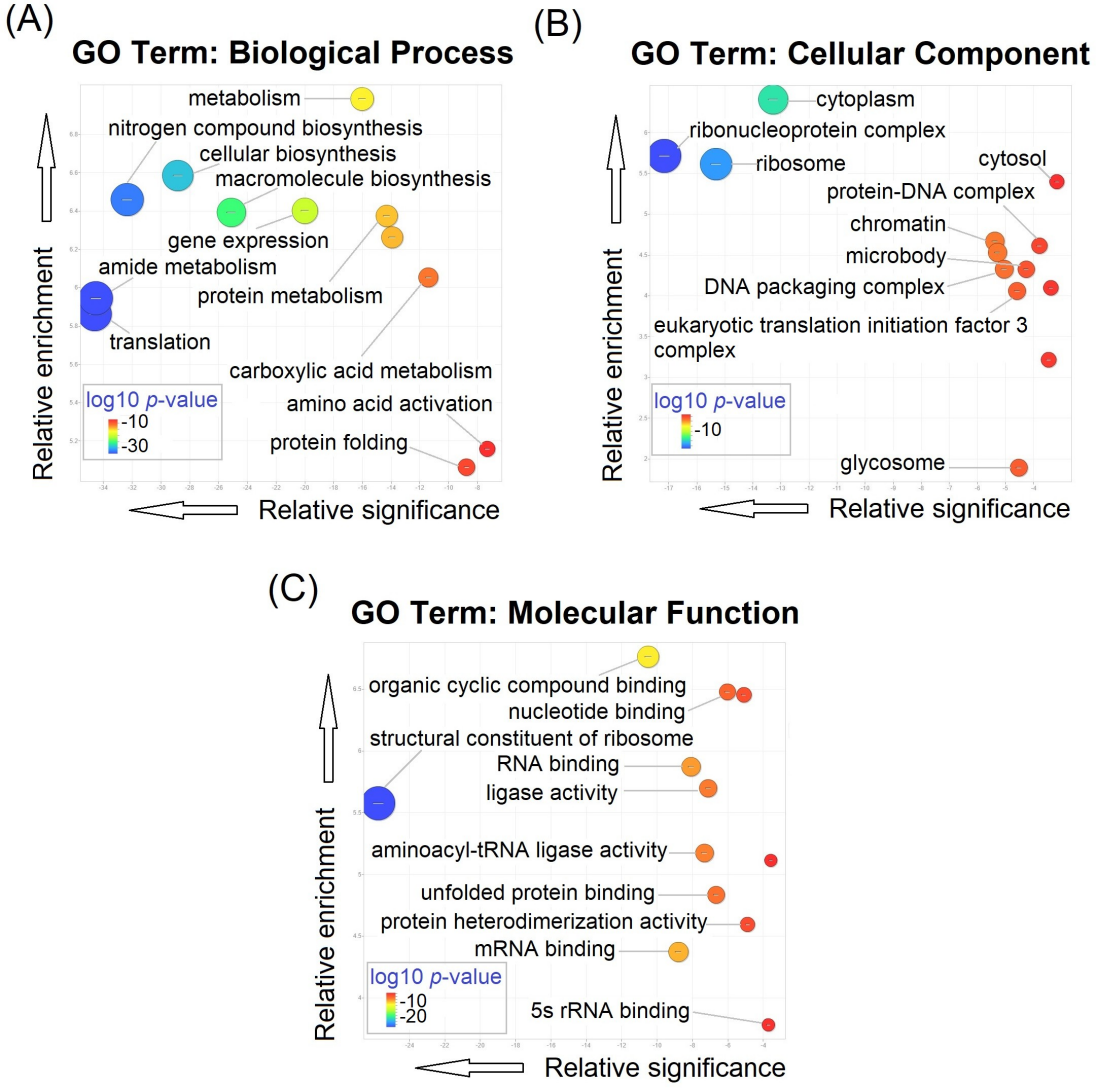
Gene Ontology Term enrichment of the 299 starvation-specific NSPs at 2 hours of starvation relative to the predicted *L*. *mexicana* whole proteome. (**A**) GO term enrichment for Biological Process (**B**) GO term enrichment for Cellular Component, and (**C**) GO term enrichment for Molecular Function. The GO terms were refined and visualised using REVIGO software.

Biological Process GO term enrichment analysis (Fig 7A) of the complete 299 statistically significant protein IDs (S3 Table) of the starvation-responsive NSPs at 2 hour duration of starvation revealed translation (P value 2.47e^-35^; 70 entries), cellular amide metabolic processes (P value 2.99e^-35^; 73 entries) and peptide biosynthetic process (P value 4.84e^-35^; 70 entries) as the most significantly enriched terms. Gene expression (P value 1.01e^-20^; 76 entries) was also among highly enriched terms. Ribonucleoprotein complex (P value 6.88e^-18^; 61 entries) and ribosome (P value 4.87e^-16^; 52 entries) were the most significantly enriched Cellular Component GO terms (Fig 7B). Similarly, Molecular Function GO term analysis (Fig 7C) revealed structural constituent of ribosome (P value 1.71e^-26^; 51 entries), organic cyclic compound binding (P value 3.14e^-11^; 127 entries), RNA binding (P value 8.35e^-9^; 43 entries), aminoacyl t-RNA ligase activity (P value 4.83e^-8^; 11 entries) and unfolded protein binding (P value 2.19e^-7^; 12 entries) as the most significantly enriched terms. The GO analyses clearly indicate the high specificity of the identified NSP towards regulation of protein synthesis in the ribosome relative to the available data of whole cell proteome of the parasite (Tritrypdb.org).

## Discussion

We have developed and applied an integrated chemical proteomics method that combines the BONCAT approach with bead-based affinity enrichment and iTRAQ quantitative proteomics MS for robust and sensitive profiling of starvation-responsive NSPs of *Leishmania*. Although the alternative, ribosome profiling [32,33] is emerging as a powerful method for global profiling of protein translation, MS-based proteomics, comparatively, provides a more direct, and therefore more reliable, readout of the cellular proteome and its changes under different perturbations [34]. Proteins are more robust during sample handling, whilst every step in the experimental protocols of ribosome profiling from cell lysis to nuclease digestion to library generation is likely to cause distortions in the data [35]. The use of translational inhibitors during ribosome profiling is also known to affect the local distribution of ribosomes on mRNAs [36]. Additionally, false readout of translation due to contaminating ribosomal RNA (rRNA) fragments is a common occurrence in ribosome profiling [37]. In a starvation condition, when the global translation levels are low, the rRNA contaminating fragments could significantly compromise the ribosome footprint sequencing space [32]. Our BONCAT-iTRAQ MS approach in *Leishmania* provides a powerful alternative to the ribosome profiling in the protozoa, and the method in *L*. *mexicana* promastigotes enabled direct profiling of the NSPs and their relative quantitative changes in abundance under starvation in a time-dependent manner.

Our initial BONCAT-iTRAQ 4-plex labelling experiments demonstrated the effectiveness of the integrated method in identifying the NSPs and quantifying their relative changes in abundance at different durations of starvation in the parasite. However, as the fold changes in protein abundances were calculated with respect to the vehicle control treatment, a second round of BONCAT-iTRAQ labelling experiments were performed to establish high-confidence starvation-responsive NSPs in the parasite. In this case, AHA-treated iTRAQ-labelled non-starved parasites were used as controls and the fold changes in protein abundances at 1 hour and 2 hour durations of starvation were calculated with respect to this control. These BONCAT-iTRAQ 3-plex labelling experiments firmly established not only the starvation-responsive NSPs but their relative quantitative changes in abundance compared to the non-starved cells at the two tested durations of starvation.

Stress conditions including nutrient deprivation can trigger initiation of endoplasmic reticulum (ER) stress responses [38]. In order to mitigate the ER stress and to re-establish the cellular homeostasis, an array of signalling pathways, collectively termed unfolded protein response (UPR), constantly surveys the conditions in the ER lumen, where misfolded proteins accumulate, and report the information to gene expression programmes [39]. Although the exact mechanisms of UPR and the key molecular players in the pathway in *Leishmania* are yet to be uncovered, our data clearly shows increased expression of several proteins that might play crucial roles in the ER stress response pathways in the parasite. For instance, the ER heat shock protein 70 (HSP70) family member chaperone BiP protein (Gene ID: LmxM.28.1200), robustly detected among the top starvation-responsive proteins, is a master regulator of ER stress response functions in higher eukaryotes [40]. BiP’s interaction with its client proteins play a vital role in the chaperoning of NSPs, and it also plays an important role in maintaining the permeability barrier of the ER lumen during protein translocation and guide the misfolded proteins for retrograde translocation to cytosol for degradation in the proteasome. Importantly, the ubiquitin-conjugating enzyme E2, putative (LmxM.04.0680), which carries out the second step in the protein ubiquitination reaction that label proteins for degradation in the proteasome [41] was also detected among the top ranking proteins. In addition to BiP, several other heat shock proteins and chaperons including HSP70, putative (Gene ID: LmxM.28.2770), HSP, putative (Gene ID: LmxM.32.2390), HSP, putative (Gene ID: LmxM.18.1370), mitochondrial precursor heat shock 70-related protein 1 (Gene ID: LmxM.29.2490) were also detected among the top starvation-responsive NSPs. The important co-chaperone, stress-inducible protein STI1 (Gene ID: LmxM.36.0070) was also detected among the top starvation responsive NSPs. In mammals, the STI1 has been recently shown to physically connect the HSP70 and HSP90 chaperons and regulate their activities by enabling efficient handover of the client proteins [42]. The STI1 protein and its interaction with HSP90 has been shown to be critical for the proliferation of both promastigote and amastigote life cycle stages of *L*. *donovani* [43]. Similarly, in *Trypanosoma cruzi* parasites, increased protein levels of the STI1 were reported in epimastigotes under nutritional stress and was found to play a role in the differentiation of the epimastigotes to metacyclic trypomastigotes [44]. Although lower in the relative expression ranking, the HSP90-family protein (also known as HSP83 in *Leishmania spp*.; Gene ID: LmxM.32.0312) was also observed among the starvation-responsive NSPs. HSP90 has been recently shown to act as a downstream client of phosphorylation-mediated cellular signalling in *L*. *donovani* [45]. Similarly, HSPs in the HSP90 foldosome complex in *L*. *donovani* were recently shown to be phosphorylated by mitogen-activated protein kinase-1 [46]. Beyond their well-established role in protein folding, HSPs and many components of the cellular protein folding machinery are found to be associated with signalling molecules including protein kinases, transcription factors and receptors and mediate their activity [47]. Important proteins that are known to facilitate or accelerate protein folding such as prefoldin subunit 3 (Gene ID: LmxM.26.1380) [48] and peptidyl-prolyl cis-trans isomerase (Gene ID: LmxM.06.0120) [49] were also observed among the top starvation-responsive NSPs. Collectively, our data indicate that the *Leishmania* parasite exerts increased expression of several protein quality control proteins to cope up with the nutritional stress in the tested durations of starvation.

Although the function of the coatomer protein complexes in *Leishmania* remain unannotated, in higher eukaryotes, components of the coatomer protein complexes mediate biosynthetic protein transport from the ER to the trans Golgi network, and in association with the ADP ribosylation factors (ARFs) play an essential role in the retrograde Golgi to ER transport of dilysine-tagged proteins [50]. The identification of coatomer subunit delta (Gene ID: LmxM.16.1180) along with ADP ribosylation factor 3 (ARF3), putative (Gene ID: LmxM.08_29.0880) among the top starvation-responsive NSPs point to the increased reliance of the parasite on proteins that mediate intracellular trafficking, secretion and vesicular transport. In higher eukaryotes, the coat protein 1 in association with ARF has been recently shown to play a critical role in the energy homeostasis and cell survival during starvation [51]. It is likely that these proteins play a role in the energy homeostasis during the short window of severe nutrient deprivation in the parasite. Several proteins involved in the energy production and conversion such as the mitochondrial protein ATP synthase epsilon chain, putative (Gene ID: LmxM.29.3600), vacuolar ATP synthase catalytic subunit A (Gene ID: LmxM.33.3670), GTP binding protein, putative (Gene ID: LmxM.27.2330), ATP synthase subunit beta (Gene ID: LmxM.25.1180), dihydrolipoamide acetyltransferase component of pyruvate dehydrogenase complex (Gene ID: LmxM.36.2660) were also among the top ranking starvation-specific NSPs detected. Increased relative expression of these proteins indicate that the parasite tries to overcome the nutritional stress by increasing the energy availability.

The observed increased expression of many essential translation factors such as eukaryotic translation initiation factor 2 subunit, putative (Gene ID: LmxM.09.1070), mitochondrial elongation factor Tu, putative (Gene ID: LmxM.18.0740), eukaryotic translation initiation factor 3 subunit b, putative (Gene ID: LmxM.17.1290), elongation initiation factor 2 alpha subunit, putative (Gene ID: LmxM.03.0980) and several translation facilitating RNA-binding proteins point to the parasite’s increased reliance on its translational machinery during starvation.

Some of the top-ranking starvation-responsive NSPs identified in this study are also known to be important from a disease-tackling and/or inhibitor development point of view. For instance, point mutations in pyridoxal kinase (Gene ID: LmxM.29.1250) has been reported to be associated with Miltefosine resistance in *L*. *major* [52]. Another important protein, activated protein kinase c receptor (LACK; Gene ID: LmxM.28.2740), has been reported to act as a T-cell epitope, and was proposed as a potential candidate for vaccine development [53]. Another top-ranking protein, tryparedoxin peroxidase (Gene ID: LmxM.15.1160), is an important enzyme the parasite relies on for detoxifying reactive oxygen species [54]. This protein has been found to be upregulated in amphotericin B-resistant isolates [55] and antimony-resistant isolates of *Leishmania spp*. [56], indicating its possible role in drug resistance. Several studies indicate that there is a complex interplay between the stress responses and drug resistance in the *Leishmania* parasites [57–59]. Other potentially important therapeutically relevant proteins include the mitogen activated protein kinase 10 (Gene ID: LmxM.10.0200) and the cell division protein kinase 2 (Gene ID: LmxM.21.1080); the former has been reported to be stage-specifically regulated in *Leishmania* with its kinase activity increasing from promastigote to amastigote conversion [60] and the latter is a protein kinase that acts as a crucial regulator of cell division cycle in the parasite [61].

Our results indicate that”translation, ribosome structure and biogenesis”, “posttranslational modifications, protein turnover and chaperons” and “energy production and conversion” were among the most representative enriched functional annotations of the NSPs identified (S2 Fig). This is in congruence with the previous finding that *Leishmania* exerts an increased level of control on translation during stress conditions [62]. A higher level control on translation is expected under starvation as translation is energetically a costly process for the cell [63], and therefore the parasite has to rely on an increased level of control on translation, and potentially posttranslational mechanisms as well, for conserving the available limited nutrient resources, and to optimise and appropriately regulate protein synthesis to avoid generating toxic protein forms. This is the first study that comprehensively and quantitatively profiled the NSPs during starvation in *Leishmania*. It is, however, important to note that despite the recent advancements in the genome sequencing of several *Leishmania* strains, a major portion of the predicted parasite proteome remain functionally unannotated and termed uncharacterised. Nevertheless, bioinformatics methods such as protein-protein interaction mapping [64], domain identification [65] and structural homology modelling [66] are making advancements in the protein functional annotation efforts. Therefore, we believe that along with future developments in more detailed functional characterisation of the *Leishmania* proteome, our results will provide additional insights into the molecular mechanisms involved in regulating the gene expression under severe starvation in the protozoan. Regulation of protein synthesis in kinetoplastids is currently poorly understood. Our method introduces a powerful platform for studying the protein synthesis in the parasites in a temporally resolved, quantitative and high-throughput manner. It is anticipated that our methodology will find wide-spread applications in the kinetoplastida parasites and in the broader area of NTD, and the results from this study will serve as a starting point for future studies to unravel the starvation-adaptation mechanisms in different life cycle stages in these parasites.

## Supporting information

S1 Fig Chemical structure of the capture reagents used. (**A**) 5-TAMRA-Alkyne used for click chemistry followed by in-gel fluorescence imaging. (**B**) Acetylene-PEG4-Biotin used for click chemistry followed by affinity enrichment and iTRAQ proteomics MS.
(TIF)

S2 Fig Functional annotation pie chart of the starvation-responsive NSPs. The complete list of starvation-responsive NSPs identified were functionally classified using the eggNOG database and the different functional categories depicted. The following letter codes were used for the functional categories in the pie chart. (F) Function unknown; (T) Translation, ribosomal structure and biogenesis; (P) Post-translational modification, protein turnover, and chaperones; (E) Energy production and conversion; (A) Amino acid transport and metabolism; (C1) Carbohydrate transport and metabolism; (C2) Coenzyme transport and metabolism; (C3) Chromatin structure and dynamics; (C4) Cytoskeleton; (I) Intracellular trafficking, secretion, and vesicular transport; (L) Lipid transport and metabolism; (N) Nucleotide transport and metabolism; (R) Replication, recombination and repair; (S) Signal transduction mechanisms; (T2) Transcription.
(TIF)

S1 Table LC-MS/MS protein identification and quantification output. The complete list of proteins identified in two replicate iTRAQ 4-plex labelling experiments along with the corrected reported intensities of the four iTRAQ channels for each protein in the two experiments following MaxQuant-Perseus database search and data processing. The corrected reporter intensities of each iTRAQ channel is presented as a fold change (FC) in log2 scale from the DMSO control 114 channel. The symbol NaN indicates a non-valid value resulting from missing reporter ion signals. T-test significant (p ≤ 0.05) entries are indicated with a + sign and only those proteins that are both significant and with 2 or more identified unique peptides were used for subsequent bioinformatic analysis.
(XLSX)

S2 Table Top-50 NSPs identified at 1 hour, 2 hour and 3 hour durations of starvation. The proteins are listed in the descending order of their observed FC in abundance values in log_2_ scale.
(PDF)

S3 Table LC-MS/MS protein identification and quantification output of starvation-responsive NSPs. The complete list of proteins identified in two replicate iTRAQ 3-plex labelling experiments along with the corrected reported intensities of the three iTRAQ channels for each protein in the two experiments following MaxQuant-Perseus database search and data processing. The corrected reporter intensities of each iTRAQ channel is presented as a fold change (FC) in log2 scale from the non-starved AHA-treated control 114 channel. The symbol NaN indicates a non-valid value resulting from missing reporter ion signals. T-test significant (p ≤ 0.05) entries are indicated with a + sign and only those proteins that are both significant and with 2 or more identified unique peptides were used for subsequent bioinformatic analysis.
(XLSX)

S4 Table Functional classification of starvation-responsive NSPs. The proteins are listed in the descending order of their observed FC in abundance values in log_2_ scale relative to the non-starved AHA-treated samples.
(PDF)
