# Supplementary figures and images for: A BONCAT-iTRAQ method enables temporally resolved quantitative profiling of newly synthesised proteins in Leishmania mexicana parasites during starvation
Source: PLoS Negl Trop Dis. 2019 Dec 19;13(12):e0007651. doi: 10.1371/journal.pntd.0007651 (PMC6939940; doi:10.1371/journal.pntd.0007651)

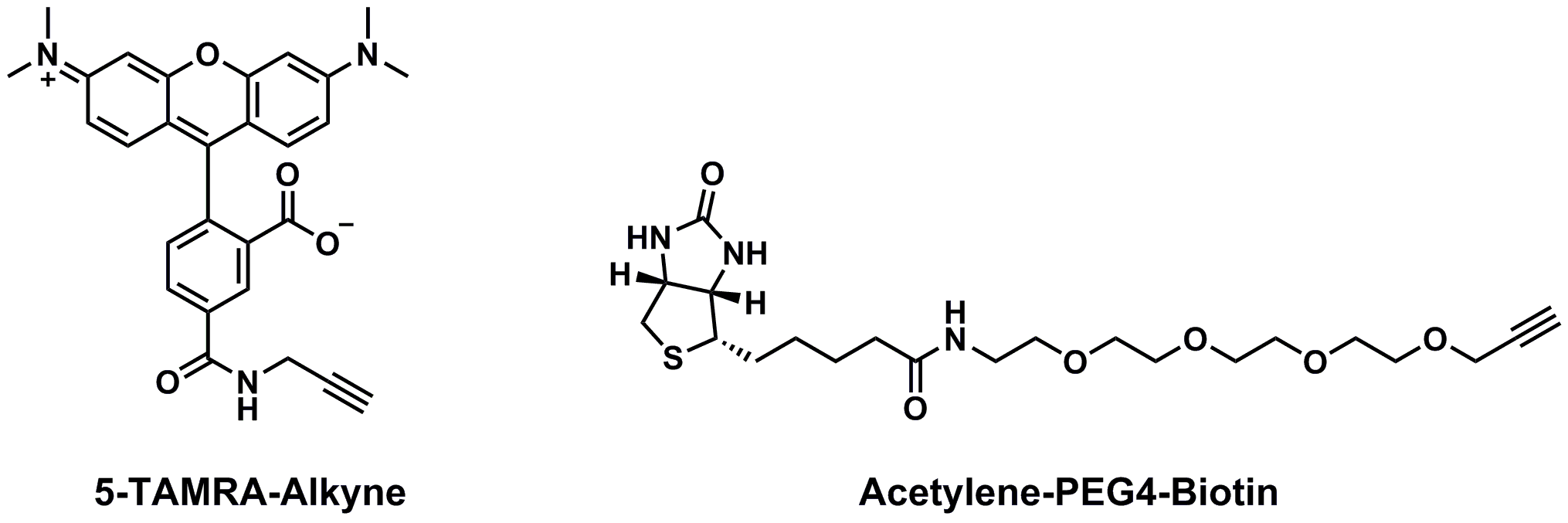

Supplement: S1 Fig — (A) 5-TAMRA-Alkyne used for click chemistry followed by in-gel fluorescence imaging. (B) Acetylene-PEG4-Biotin used for click chemistry followed by affinity enrichment and iTRAQ proteomics MS. (TIF) [file pntd.0007651.s001.tif]

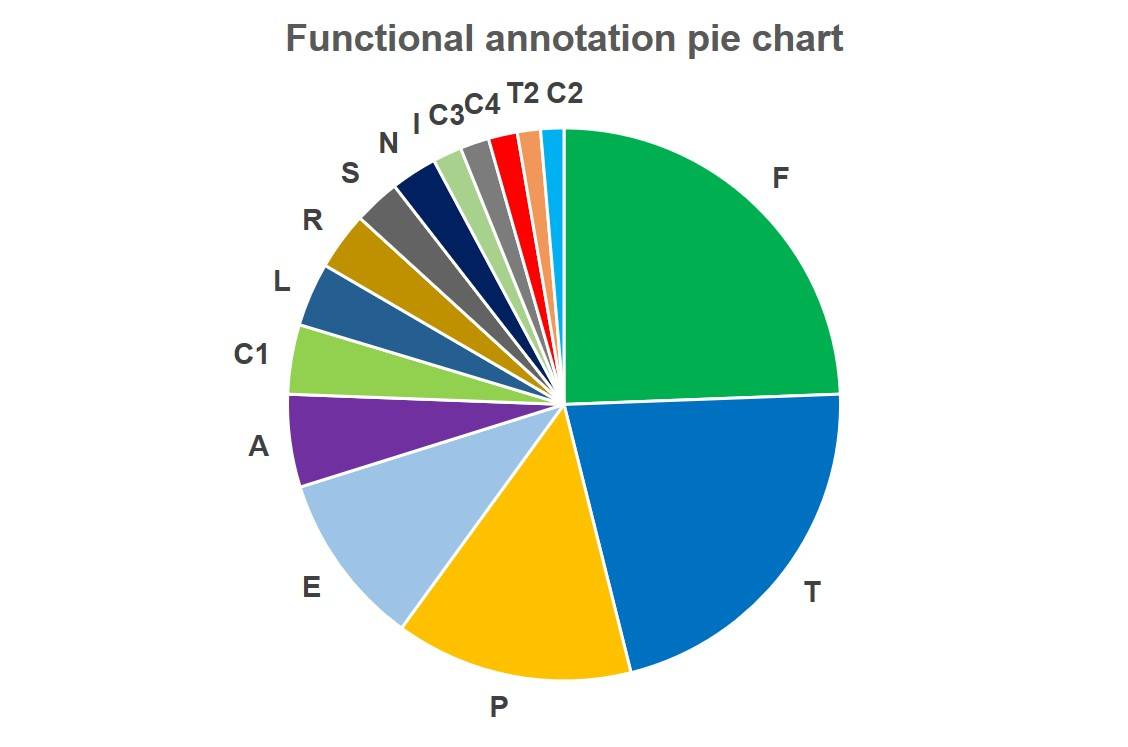

Supplement: S2 Fig — The complete list of starvation-responsive NSPs identified were functionally classified using the eggNOG database and the different functional categories depicted. The following letter codes were used for the functional categories in the pie chart. (F) Function unknown; (T) Translation, ribosomal structure and biogenesis; (P) Post-translational modification, protein turnover, and chaperones; (E) Energy production and conversion; (A) Amino acid transport and metabolism; (C1) Carbohydrate transport and metabolism; (C2) Coenzyme transport and metabolism; (C3) Chromatin structure and dynamics; (C4) Cytoskeleton; (I) Intracellular trafficking, secretion, and vesicular transport; (L) Lipid transport and metabolism; (N) Nucleotide transport and metabolism; (R) Replication, recombination and repair; (S) Signal transduction mechanisms; (T2) Transcription. (TIF) [file pntd.0007651.s002.tif]
