# Supplementary material for: A BONCAT-iTRAQ method enables temporally resolved quantitative profiling of newly synthesised proteins in Leishmania mexicana parasites during starvation
Source: PLoS Negl Trop Dis. 2019 Dec 19;13(12):e0007651. doi: 10.1371/journal.pntd.0007651 (PMC6939940; doi:10.1371/journal.pntd.0007651)
Supplement: S2 Table — The proteins are listed in the descending order of their observed FC in abundance values in log2 scale. (PDF) [file pntd.0007651.s004.pdf]

S2 Table. Top-50 NSPs at 1, 2 and 3 hour durations of starvation

1 hour starvation

Protein name	Gene ID[a]	Protein IDs[b]	Mol. weight [kDa]	-Log(P-value)	Log_2 FC	Functional annotation[c]
Kinetoplastid membrane protein-11	LmxM.34.2221	E9B6A2	11.22	1.747435	4.75655174	Function unknown
Elongation initiation factor 2 alpha subunit,putative	LmxM.03.0980	E9AJY2	46.623	2.219643	4.39524913	Translation, ribosomal structure and biogenesis
Glyceraldehyde-3-phosphate dehydrogenase	LmxM.29.2980	E9B170	39.123	2.547562	3.87727702	Carbohydrate transport and metabolism
Eukaryotic translation initiation factor 5A	LmxM.25.0720	E9AXF0	17.828	3.813115	3.83499169	Translation, ribosomal structure and biogenesis
GDP-mannose pyrophosphorylase	LmxM.23.0110	E9AW11	41.49	1.55459	3.7873385	Cell wall/membrane/envelope biogenesis
Putative 60S ribosomal protein L22	LmxM.36.3270	E9ATA6	15.074	3.863737	3.76711023	Translation, ribosomal structure and biogenesis
Putative 60S acidic ribosomal protein P2	LmxM.29.3730	E9B1E7	10.627	2.262506	3.74683046	Translation, ribosomal structure and biogenesis
Tubulin beta chain	LmxM.32.0792	E9AMJ9	49.723	3.188093	3.59441948	Cytoskeleton
Tryparedoxin peroxidase	LmxM.15.1160	E9AQA6	22.21	2.248598	3.5235759	Post-translational modification, protein turnover, and chaperones
Putative orotidine-5-phosphate decarboxylase/orotate phosphoribosyltransferase, putative	LmxM.16.0550	E9AQL3	49.674	1.355092	3.51732945	Nucleotide transport and metabolism
RNA-binding protein, putative, UPB2	LmxM.25.0500	E9AXC7	19.012	2.688037	3.45580649	Function unknown
60S ribosomal protein L6	LmxM.15.1000	E9AQ99	21.037	1.36506	3.45497203	Translation, ribosomal structure and biogenesis
Ubiquitin-fusion protein	LmxM.30.1900	E9B1Y9	8.865	2.410696	3.38150311	Translation, ribosomal structure and biogenesis

Histone H2A	LmxM.08_29.1740	E9ALP9	13.96	3.177759	3.31244648	Chromatin structure and dynamics
Transaldolase	LmxM.16.0760	E9AQN5	36.976	1.985447	3.23070216	Carbohydrate transport and metabolism
S-adenosylmethionine synthase	LmxM.29.3500	E9B1C6	43.128	1.789644	3.2182833	Coenzyme transport and metabolism
Activated protein kinase c receptor (LACK)	LmxM.28.2740	E8NHN2	34.402	2.47599	3.21782923	Function unknown
Biotin/lipoate protein ligase-like protein	LmxM.30.1070	E9B1Q4	28.493	1.769636	3.19411302	Coenzyme transport and metabolism
Tubulin alpha chain	LmxM.13.0390	E9AP62	60.184	3.635642	3.14616191	Cytoskeleton
Uncharacterized protein	LmxM.34.4470	E9B6X6	21.837	1.962042	3.14583993	Function unknown
Putative RNA binding protein	LmxM.31.0750	E9B2J9	25.249	2.270212	3.11596882	Function unknown
Putative ribosomal protein S29	LmxM.28.2460	E9B042	6.6828	2.352051	3.10722029	Translation, ribosomal structure and biogenesis
Putative 60S ribosomal protein L17	LmxM.24.0040	E9AWJ4	19.083	1.577719	3.09212303	Translation, ribosomal structure and biogenesis
40S ribosomal protein S2	LmxM.31.0450	E9B2G6	28.634	4.029102	3.08492601	Translation, ribosomal structure and biogenesis
Peroxidoxin (Tryparedoxin peroxidase)	LmxM.23.0040	E9AW04	25.373	4.706448	3.07832205	Post-translational modification, protein turnover, and chaperones
Uncharacterized protein	LmxM.19.1160	E9AS67	41.311	2.051207	3.03751564	Function unknown
Putative ATP-dependent RNA helicase	LmxM.34.3100	E9B6I9	100.88	2.878875	3.02035427	Replication, recombination and repair
Uncharacterized protein	LmxM.13.0450	E9AP69	13.323	5.551102	3.01243997	Function unknown
Chaperonin HSP60, mitochondrial	LmxM.36.2020	E9ASX7	60.305	3.341937	3.00977767	Post-translational modification, protein turnover, and chaperones
Putative paraflagellar rod protein 1D	LmxM.08_29.1750	E9ALP8	68.976	1.632049	2.9794687	Function unknown
Uncharacterized protein	LmxM.08_29.0320	E9AM47	36.554	2.03952	2.9662962	Function unknown
Putative T-complex protein 1, theta subunit	LmxM.36.6910	E9AUC7	58.239	0.978223	2.96609175	Post-translational modification, protein turnover, and chaperones

Putative 60S ribosomal protein L10	LmxM.04.0950	E9AK58	24.557	1.442417	2.96080458	Translation, ribosomal structure and biogenesis
Elongation factor 1-alpha	LmxM.17.0080	E9ARD0	49.176	2.355406	2.95602143	Translation, ribosomal structure and biogenesis
Peptidyl-prolyl cis-trans isomerase	LmxM.25.0910	E9AXG9	18.794	2.077774	2.95539153	Post-translational modification, protein turnover, and chaperones
Transitional endoplasmic reticulum ATPase,putative	LmxM.36.1370	E9ASQ6	86.887	2.403551	2.93849766	Post-translational modification, protein turnover, and chaperones
Putative malic enzyme	LmxM.24.0770	E9AWR8	63.357	1.008271	2.92175823	Energy production and conversion
Elongation factor 2	LmxM.36.0180	E9ASD6	94.056	3.542708	2.91629946	Translation, ribosomal structure and biogenesis
Succinate--CoA ligase [ADP-forming] subunit alpha, mitochondrial	LmxM.25.2140	E9AXU7	30.944	1.217772	2.90381026	Energy production and conversion
Putative ribosomal protein L38	LmxM.03.0250	E9AJR0	9.4541	1.740528	2.8976686	Translation, ribosomal structure and biogenesis
Putative 60S ribosomal protein L10a	LmxM.18.0620	E9ARK0	24.578	1.39099	2.88952076	Translation, ribosomal structure and biogenesis
Uncharacterized protein	LmxM.23.0080	E9AW08	48.444	3.694215	2.88214278	Function unknown
Putative cysteine synthase	LmxM.36.3590	E9ATD8	35.418	1.46572	2.85339224	Amino acid transport and metabolism
Protein disulfide-isomerase	LmxM.36.6940	E9AUD1	52.188	1.55617	2.8510716	Post-translational modification, protein turnover, and chaperones
Actin	LmxM.04.1230	E9AKA4	40.483	1.416989	2.84424388	Cytoskeleton
Putative translation initiation factor	LmxM.17.1290	E9ARA1	80.717	1.763449	2.8438468	Translation, ribosomal structure and biogenesis
Putative 60S ribosomal protein	LmxM.26.2330	E9AYL4	15.191	1.875178	2.84156954	Translation, ribosomal structure and biogenesis
Phosphotransferase	LmxM.21.0250	E9AUZ2	51.67	2.630802	2.83974576	Carbohydrate transport and metabolism
I/6 autoantigen-like protein	LmxM.22.1460	E9AVX8	22.879	1.039093	2.81933129	Function unknown
Putative 40S ribosomal protein S18	LmxM.36.0930	E9ASL3	17.368	2.727962	2.81688404	Translation, ribosomal structure and biogenesis

2 hours of starvation

**Protein name**	Gene ID[a]	Protein IDs[b]	Mol. weight [kDa]	-Log(P-value)	Log_2 FC	Functional annotation[c]
Elongation initiation factor 2 alpha subunit,putative	LmxM.03.0980	E9AJY2	46.623	3.316291	3.95372629	Translation, ribosomal structure and biogenesis
Kinetoplastid membrane protein-11	LmxM.34.2221	E9B6A2	11.22	2.046619	3.62992346	Function unknown
Tubulin beta chain	LmxM.32.0792	E9AMJ9	49.723	3.686411	3.5268867	Cytoskeleton
RNA-binding protein, putative	LmxM.25.0500	E9AXC7	19.012	2.51055	3.38392687	Function unknown
Glyceraldehyde-3-phosphate dehydrogenase	LmxM.29.2980	E9B170	39.123	2.269162	3.35062122	Carbohydrate transport and metabolism
Biotin/lipoate protein ligase-like protein	LmxM.30.1070	E9B1Q4	28.493	1.707647	3.319175	Coenzyme transport and metabolism
Tryparedoxin peroxidase	LmxM.15.1160	E9AQA6	22.21	2.19709	3.28370428	Post-translational modification, protein turnover, and chaperones
60S ribosomal protein L6	LmxM.15.1000	E9AQ99	21.037	1.298993	3.19907737	Translation, ribosomal structure and biogenesis
Putative 60S ribosomal protein L22	LmxM.36.3270	E9ATA6	15.074	1.647716	3.13306046	Translation, ribosomal structure and biogenesis
Eukaryotic translation initiation factor 5A	LmxM.25.0720	E9AXF0	17.828	2.295076	3.0681839	Translation, ribosomal structure and biogenesis
Putative ATP-dependent RNA helicase	LmxM.34.3100	E9B6I9	100.88	2.399055	3.05207014	Replication, recombination and repair
Activated protein kinase c receptor (LACK)	LmxM.28.2740	E8NHN2	34.402	1.311391	3.03074849	Function unknown
Uncharacterized protein	LmxM.13.0450	E9AP69	13.323	2.062774	2.97216988	Function unknown
Putative 60S ribosomal protein L17	LmxM.24.0040	E9AWJ4	19.083	1.89648	2.95896637	Translation, ribosomal structure and biogenesis
IgE-dependent histamine-releasing factor,putative	LmxM.24.1500	E9AWZ6	19.317	1.422718	2.95895326	Function unknown

Putative orotidine-5-phosphate decarboxylase/orotate phosphoribosyltransferase, putative	LmxM.16.0550	E9AQL3	49.674	2.208132	2.94959939	Nucleotide transport and metabolism
Transaldolase OS=Leishmania mexicana	LmxM.16.0760	E9AQN5	36.976	1.138446	2.90897417	Carbohydrate transport and metabolism
Putative ribosomal protein L38	LmxM.03.0250	E9AJR0	9.4541	1.212759	2.88025892	Translation, ribosomal structure and biogenesis
Tubulin alpha chain	LmxM.13.0280	E9AP62	60.184	3.803246	2.84298086	Cytoskeleton
Chaperonin HSP60, mitochondrial	LmxM.36.2020	E9ASX7	60.305	2.844623	2.81874132	Post-translational modification, protein turnover, and chaperones
I/6 autoantigen-like protein	LmxM.22.1460	E9AVX8	22.879	0.766946	2.77909732	Function unknown
S-adenosylmethionine synthase	LmxM.29.3500	E9B1C6	43.128	1.503425	2.77750826	Coenzyme transport and metabolism
Putative RNA binding protein	LmxM.31.0750	E9B2J9	25.249	2.885225	2.76665711	Function unknown
Elongation factor 2	LmxM.36.0180	E9ASD6	94.056	3.666589	2.75053024	Translation, ribosomal structure and biogenesis
Peroxidoxin (Tryparedoxin peroxidase)	LmxM.23.0040	E9AW04	25.373	2.185045	2.75020289	Post-translational modification, protein turnover, and chaperones
40S ribosomal protein S2	LmxM.31.0450	E9B2G6	28.634	2.564065	2.73739183	Translation, ribosomal structure and biogenesis
Putative 60S ribosomal protein L10a	LmxM.18.0620	E9ARK0	24.578	1.541825	2.73458493	Translation, ribosomal structure and biogenesis
Succinate--CoA ligase [ADP-forming] subunit alpha, mitochondrial	LmxM.25.2140	E9AXU7	30.944	1.828058	2.72421157	Energy production and conversion
ATP synthase subunit beta	LmxM.25.1180	E9AXJ7	53.07	4.279773	2.71239352	Energy production and conversion
Actin	LmxM.04.1230	E9AKA4	40.483	1.231243	2.70171773	Cytoskeleton
Putative 60S acidic ribosomal protein P2	LmxM.29.3730	E9B1E7	10.627	1.484067	2.69413793	Translation, ribosomal structure and biogenesis
Putative 60S ribosomal protein L10	LmxM.04.0750	E9AK58	24.557	1.468084	2.6781714	Translation, ribosomal structure and biogenesis
Uncharacterized protein	LmxM.19.1160	E9AS67	41.311	2.673168	2.67754412	Function unknown

Mannose-1-phosphate guanyltransferase	LmxM.23.0110	E9AW11	41.49	3.57882	2.67233467	Cell wall/membrane/envelope biogenesis
Putative heat-shock protein hsp70	LmxM.28.2770	E9B099	71.24	4.306776	2.64959431	Post-translational modification, protein turnover, and chaperones
Uncharacterized protein	LmxM.34.4470	E9B6X6	21.837	2.102834	2.6420002	Function unknown
NAD-specific glutamate dehydrogenase	LmxM.15.1010	E9AQA0	114.65	4.633952	2.63021708	Amino acid transport and metabolism
40S ribosomal protein S18	LmxM.36.0930	E9ASL3	17.368	1.692427	2.62145591	Translation, ribosomal structure and biogenesis
Phosphotransferase	LmxM.21.0250	E9AUZ2	51.67	2.317513	2.60266876	Carbohydrate transport and metabolism
Enolase	LmxM.14.1160	E9APW3	46.145	4.005362	2.56035161	Carbohydrate transport and metabolism
Putative ATPase alpha subunit	LmxM.05.0500	E8NHQ7	62.599	7.211016	2.55363095	Energy production and conversion
Putative cysteine synthase	LmxM.36.3590	E9ATD8	35.418	0.84883	2.54563868	Amino acid transport and metabolism
Elongation factor 1-alpha	LmxM.17.0080	E9ARD0	49.176	2.05855	2.5268544	Translation, ribosomal structure and biogenesis
Uncharacterized protein	LmxM.23.0080	E9AW08	48.444	1.794491	2.52217257	Function unknown
Putative ribosomal protein S6	LmxM.15.1470	E9AQE3	13.516	2.347715	2.51198816	Translation, ribosomal structure and biogenesis
T-complex protein 1 subunit eta	LmxM.34.3860	E9B6R4	61.731	1.806438	2.50944209	Post-translational modification, protein turnover, and chaperones
40S ribosomal protein S24	LmxM.36.2860	E9AT64	15.752	2.824758	2.50235701	Translation, ribosomal structure and biogenesis
Peptidyl-prolyl cis-trans isomerase	LmxM.25.0910	E9AXG9	18.794	1.968066	2.50043511	Post-translational modification, protein turnover, and chaperones
Uncharacterized protein	LmxM.08.1100	E9AMI0	42.21	2.080958	2.47440791	Function unknown
40S ribosomal protein S3a	LmxM.34.0400	E8NHI0	30.036	1.97874	2.45464265	Translation, ribosomal structure and biogenesis

3 hours of starvation

**Protein name**	Gene ID[a]	Protein IDs[b]	Mol. weight [kDa]	-Log (P-value)	Log_2 FC	Functional annotation[c]
Putative 60S ribosomal protein L22	LmxM.36.3270	E9ATA6	15.074	2.220319	2.90907776	Translation, ribosomal structure and biogenesis
Putative 60S ribosomal protein L35	LmxM.26.2330	E9AYL4	15.191	1.625769	2.14794374	Translation, ribosomal structure and biogenesis
Uncharacterized protein	LmxM.23.0080	E9AW08	48.444	0.962109	2.03158337	Function unknown
I/6 autoantigen-like protein	LmxM.22.1460	E9AVX8	22.879	0.589846	2.00453371	Function unknown
Histone H2A	LmxM.08_29.1740	E9ALP9	13.96	3.448067	1.90597433	Chromatin structure and dynamics
Putative 60S ribosomal protein L17	LmxM.24.0040	E9AWJ4	19.083	1.799147	1.86069119	Translation, ribosomal structure and biogenesis
Kinetoplastid membrane protein-11	LmxM.34.2221	E9B6A2	11.22	1.111731	1.85669327	Function unknown
RNA-binding protein, putative	LmxM.25.0500	E9AXC7	19.012	2.12655	1.72728658	Function unknown
Activated protein kinase c receptor (LACK)	LmxM.28.2740	E8NHN2	34.402	1.605377	1.67772818	Function unknown
Uncharacterized protein	LmxM.13.0450	E9AP69	13.323	1.711689	1.66980648	Function unknown
Aconitate hydratase	LmxM.18.0510	E9ARI8	97.47	1.212084	1.60593414	Energy production and conversion
Glyceraldehyde-3-phosphate dehydrogenase	LmxM.29.2980	E9B170	39.123	1.261743	1.45159495	Carbohydrate transport and metabolism
Putative 40S ribosomal protein S18	LmxM.36.0930	E9ASL3	17.368	3.556283	1.40759438	Translation, ribosomal structure and biogenesis
Putative 40S ribosomal protein S17	LmxM.28.2555	E9B078	16.408	2.906702	1.39721394	Translation, ribosomal structure and biogenesis
Peroxidoxin (Tryparedoxin peroxidase)	LmxM.23.0040	E9AW04	25.373	1.503624	1.38261449	Post-translational modification, protein turnover, and chaperones
Uncharacterized protein	LmxM.19.1160	E9AS67	41.311	1.012741	1.36713272	Function unknown
Putative 40S ribosomal protein S13	LmxM.19.0390	E9ARZ1	17.471	0.94141	1.34424314	Translation, ribosomal structure and biogenesis

S-adenosylmethionine synthase	LmxM.29.3500	E9B1C6	43.128	1.077107	1.32698098	Coenzyme transport and metabolism
60S ribosomal protein L18a	LmxM.34.0600	E9B5U1	20.779	2.718778	1.30531067	Translation, ribosomal structure and biogenesis
Elongation initiation factor 2 alpha subunit,putative	LmxM.03.0980	E9AJY2	46.623	1.850587	1.29416895	Translation, ribosomal structure and biogenesis
IgE-dependent histamine-releasing factor,putative	LmxM.24.1500	E9AWZ6	19.317	0.548691	1.26862192	Function unknown
60S ribosomal protein L11	LmxM.04.0470	E9AK29	21.675	0.973593	1.25625044	Translation, ribosomal structure and biogenesis
Transitional endoplasmic reticulum ATPase,putative	LmxM.36.1370	E9ASQ6	86.887	1.157327	1.23587134	Post-translational modification, protein turnover, and chaperones
Elongation factor 2	LmxM.36.0180	E9ASD6	94.056	2.882916	1.22415942	Translation, ribosomal structure and biogenesis
Uncharacterized protein	LmxM.33.2580	E9B549	22.572	2.554125	1.22320408	Function unknown
Tryparedoxin peroxidase	LmxM.15.1160	E9AQA6	22.21	0.81126	1.21154216	Post-translational modification, protein turnover, and chaperones
Putative ribosomal protein L27	LmxM.31.2710	E9B350	15.43	0.713048	1.19113594	Translation, ribosomal structure and biogenesis
Elongation factor-1 gamma	LmxM.09.0970	E9AMU9	46.201	1.806407	1.15208495	Translation, ribosomal structure and biogenesis
Putative T-complex protein 1, theta subunit	LmxM.36.6910	E9AUC7	58.239	0.909012	1.14306244	Post-translational modification, protein turnover, and chaperones
Tubulin beta chain	LmxM.32.0792	E9AMJ9	49.723	2.471681	1.13462615	Cytoskeleton
Uncharacterized protein	LmxM.08_29.0320	E9AM47	36.554	0.691269	1.13227007	Function unknown
Eukaryotic translation initiation factor 3 subunit b	LmxM.17.1290	E9ARA1	80.717	2.336334	1.10621524	Translation, ribosomal structure and biogenesis
Putative ribosomal protein L3	LmxM.33.2870	E9B578	47.543	5.163474	1.09349465	Translation, ribosomal structure and biogenesis
Ribosomal protein L15	LmxM.34.1910	E9B668	24.029	0.902702	1.08980501	Translation, ribosomal structure and biogenesis
Splicing factor ptsr1-like protein	LmxM.07.0870	E9ALA4	41.683	2.166786	1.08819747	RNA processing and modification

Putative 60S Ribosomal protein L36	LmxM.34.1920	E9B669	11.936	1.333709	1.08030617	Translation, ribosomal structure and biogenesis
Peptidyl-prolyl cis-trans isomerase	LmxM.25.0910	E9AXG9	18.794	1.43647	1.06882414	Post-translational modification, protein turnover, and chaperones
Mannose-1-phosphate guanyltransferase	LmxM.23.0110	E9AW11	41.49	1.080179	1.06434092	Cell wall/membrane/envelope biogenesis
Putative ribosomal protein S29	LmxM.28.2205	E9B042	6.6828	1.537321	1.05228376	Translation, ribosomal structure and biogenesis
Putative ATPase alpha subunit	LmxM.05.0500	E8NHQ7	62.599	1.379252	1.04632518	Energy production and conversion
Putative orotidine-5-phosphate decarboxylase/orotate phosphoribosyltransferase, putative	LmxM.16.0550	E9AQL3	49.674	1.688768	1.04458764	Nucleotide transport and metabolism
Putative ribosomal protein L38	LmxM.03.0250	E9AJR0	9.4541	2.758792	1.04265475	Translation, ribosomal structure and biogenesis
60S ribosomal protein L32	LmxM.34.2050	E9B683	15.336	0.82952	1.03582081	Translation, ribosomal structure and biogenesis
Putative RNA binding protein	LmxM.31.0750	E9B2J9	25.249	1.250823	1.03306028	Function unknown
Chaperonin HSP60, mitochondrial	LmxM.36.2020	E9ASX7	60.305	3.497159	0.99260774	Post-translational modification, protein turnover, and chaperones
ATP synthase subunit beta	LmxM.25.1180	E9AXJ7	53.07	2.304336	0.98296416	Energy production and conversion
Fructose-bisphosphate aldolase	LmxM.36.1260	E9ASP6	40.833	3.037292	0.95275888	Carbohydrate transport and metabolism
40S ribosomal protein S8	LmxM.24.2070	E9AX53	24.686	0.620346	0.94981696	Translation, ribosomal structure and biogenesis
RuvB-like helicase	LmxM.33.2610	E9B552	53.717	0.616864	0.89103246	Transcription
Putative ribosomal protein S20	LmxM.28.1010	E9AZR7	13.011	1.102122	0.88808432	Translation, ribosomal structure and biogenesis

The Top-50 proteins that were identified and differentially expressed at 1,2 and 3 hour durations of starvation in two replicates with significant T-Test values (P≤0.05) are presented.

[a]Gene ID according to the GeneDB: The Sanger Institute Pathogen Genomics Database (www.genedb.org).

[b]Protein ID according to the Universal Protein Resource (UniProt) (www.uniprot.org).

[c]Functional classification determined by eggNOG database.
