# Supplementary material for: A BONCAT-iTRAQ method enables temporally resolved quantitative profiling of newly synthesised proteins in Leishmania mexicana parasites during starvation
Source: PLoS Negl Trop Dis. 2019 Dec 19;13(12):e0007651. doi: 10.1371/journal.pntd.0007651 (PMC6939940; doi:10.1371/journal.pntd.0007651)
Supplement: S4 Table — The proteins are listed in the descending order of their observed FC in abundance values in log2 scale relative to the non-starved AHA-treated samples. (PDF) [file pntd.0007651.s006.pdf]

S4 Table. Starvation-responsive NSPs of *Leishmania mexicana* promastigotes

Protein name	Gene ID[a]	Protein ID[b]	Mol. weight [kDa]	-Log (P-value)	log_2 FC	Functional annotation[c]
Putative nuclear transport factor 2	LmxM.10.0850	E9AN95	13.957	1.524454	3.864078	Intracellular trafficking, secretion, and vesicular transport
Coatomer subunit delta	LmxM.16.1180	E9AQS9	58.102	2.380287	3.728916	Intracellular trafficking, secretion, and vesicular transport
Prefoldin subunit 3	LmxM.26.1380	E9AYB7	22.19	1.141505	3.694739	Function unknown
Cofilin-like protein	LmxM.08_29.0510	E9AM28	15.689	4.076255	3.628612	Function unknown
Heat shock protein Hsp20, putative	LmxM.31.2260	E9B307	20.273	1.685165	3.61035	Function unknown
Putative ATP synthase, epsilon chain	LmxM.29.3600	E9B1D4	19.986	2.087189	3.591015	Lipid transport and metabolism
Putative ADP ribosylation factor 3	LmxM.08_29.0880	E9ALY6	20.142	1.512733	3.562658	Function unknown
IgE-dependent histamine-releasing factor,putative	LmxM.24.1500	E9AWZ6	19.317	2.06034	3.337076	Function unknown
Putative 60S acidic ribosomal protein P2	LmxM.03.0430	E9AJS6	9.7078	1.789937	3.321424	Function unknown
ALBA-domain protein 1	LmxM.13.0450	E9AP69	13.323	1.991815	3.159431	Function unknown
RNA-binding protein, putative, UPB2	LmxM.25.0500	E9AXC7	19.012	1.696032	3.092635	Function unknown
Uncharacterized protein	LmxM.36.4460	E9ATN0	17.317	3.152862	3.065769	Function unknown
Putative oxidoreductase	LmxM.36.4170	E9ATK1	36.277	1.539332	3.057409	Function unknown
Putative 60S ribosomal protein L34	LmxM.36.3740	E9ATF6	19.351	1.728365	3.052743	Translation, ribosomal structure and biogenesis
Prostaglandin f2-alpha synthase	LmxM.30.2150	E9B215	31.862	3.589629	3.041707	Function unknown
Peptidyl-prolyl cis-trans isomerase	LmxM.06.0120	E9AKP0	20.433	2.08882	2.976228	Post-translational modification, protein turnover, and chaperones
Uncharacterized protein	LmxM.25.1080	E9AXI7	47.534	0.810798	2.965599	Function unknown
Putative Pyridoxal kinase	LmxM.29.1250	E9B0Q4	33.137	2.246632	2.945873	Coenzyme transport and metabolism

Putative ribosomal protein L38	LmxM.03.0250	E9AJR0	9.4541	2.839185	2.928729	Translation, ribosomal structure and biogenesis
Putative 60S ribosomal protein L12	LmxM.24.2210	E9AX67	17.538	3.38222	2.920702	Translation, ribosomal structure and biogenesis
60S acidic ribosomal protein P2	LmxM.15.1203	E9AQB1	11	2.470133	2.918778	Function unknown
Putative ribosomal protein S6 (Nph2/rs6-like protein)	LmxM.15.1470	E9AQE3	13.516	1.944989	2.902703	Translation, ribosomal structure and biogenesis
Dihydrolipoamide acetyltransferase component of pyruvate dehydrogenase complex	LmxM.36.2660	E9AT43	48.774	3.494244	2.895743	Energy production and conversion
Putative la RNA binding protein	LmxM.21.0540	E9AV21	37.206	2.536296	2.867384	Transcription
Uncharacterized protein	LmxM.25.0540	E9AXD1	32.428	3.032965	2.785401	Function unknown
Putative RNA-binding protein	LmxM.27.2100	E9AZB6	46.766	3.74254	2.773409	Function unknown
Putative small GTP-binding protein Rab11	LmxM.10.0910	E9ANA1	23.358	2.427718	2.753845	Function unknown
Putative ubiquitin-conjugating enzyme e2	LmxM.04.0680	E9AK51	17.05	3.776182	2.646444	Post-translational modification, protein turnover, and chaperones
Putative 60S ribosomal protein L22	LmxM.36.3270	E9ATA6	15.074	1.676263	2.642666	Translation, ribosomal structure and biogenesis
Vacuolar ATP synthase catalytic subunit A,putative	LmxM.33.3670	E9B5F8	67.812	2.9825	2.630277	Energy production and conversion
Eukaryotic translation initiation factor 3 subunit h	LmxM.07.0640	E9AL77	38.015	0.685132	2.627706	Translation, ribosomal structure and biogenesis
Uncharacterized protein	LmxM.19.1160	E9AS67	41.311	1.905864	2.609281	Function unknown
Replication protein A subunit	LmxM.28.1820	E9B001	52.386	2.052266	2.59775	Replication, recombination and repair
Putative mitogen-activated protein kinase	LmxM.10.0200	E9AN28	46.316	1.497464	2.590533	Signal transduction mechanisms
Aconitate hydratase	LmxM.18.0510	E9ARI8	97.47	1.264582	2.570808	Energy production and conversion
Eukaryotic translation initiation factor 3 subunit k	LmxM.31.2180	E9B2Z9	26.197	1.313855	2.566463	Translation, ribosomal structure and biogenesis
Peroxidoxin	LmxM.23.0040	E9AW04	25.373	2.154723	2.559911	Post-translational modification, protein turnover, and chaperones

Nonspecific nucleoside hydrolase (Fragment)	LmxM.18.1580	E9ARU2	32.205	0.824381	2.559882	Nucleotide transport and metabolism
Putative eukaryotic translation initiation factor 2 subunit	LmxM.09.1070	E9AMV9	52.548	3.774545	2.541689	Translation, ribosomal structure and biogenesis
Uncharacterized protein	LmxM.31.0840	E9B2K8	56.745	2.784268	2.537643	Function unknown
40S ribosomal protein S24	LmxM.36.2860	E9AT64	15.752	2.233523	2.533615	Translation, ribosomal structure and biogenesis
Putative elongation factor Tu	LmxM.18.0740	E9ARL1	51.682	1.086995	2.514242	Translation, ribosomal structure and biogenesis
Obg-like ATPase 1	LmxM.27.2330	E9AZE0	44.003	1.592856	2.504489	Translation, ribosomal structure and biogenesis
Putative 40S ribosomal protein S15A	LmxM.11.1190	E9ALP4	14.696	1.954762	2.496949	Translation, ribosomal structure and biogenesis
Putative NADH-dependent fumarate reductase	LmxM.34.1180	E9B5Z7	123.03	1.340842	2.472113	Energy production and conversion
Ribonucleoside-diphosphate reductase	LmxM.28.0890	E9AZQ5	90.849	1.557454	2.463399	Nucleotide transport and metabolism
Putative glucose-regulated protein 78	LmxM.28.1200	E9AZT9	71.792	2.376916	2.462734	Post-translational modification, protein turnover, and chaperones
Putative 60S ribosomal protein L19	LmxM.06.0410	E9AKR8	28.202	2.200114	2.459422	Translation, ribosomal structure and biogenesis
Putative translation initiation factor	LmxM.17.1290	E9ARA1	80.717	1.528962	2.44446	Translation, ribosomal structure and biogenesis
Malic enzyme	LmxM.24.0761	E9AWR7	62.562	1.828945	2.43674	Energy production and conversion
Putative ABC transporter	LmxM.08_29.0620	E9AM16	206.85	1.650155	2.388837	Defense mechanisms
60S ribosomal protein L6	LmxM.15.1000	E9AQ99	21.037	2.759018	2.352065	Translation, ribosomal structure and biogenesis
Putative 60S ribosomal protein L23	LmxM.34.3790	E9B6Q7	14.956	1.673758	2.341008	Translation, ribosomal structure and biogenesis
3-ketoacyl-CoA thiolase-like protein	LmxM.23.0690	E9AW84	47.372	2.64339	2.334126	Lipid transport and metabolism
PAB1-binding protein , putative	LmxM.10.1110	E9ANC1	56.911	1.710827	2.32877	Function unknown

Transaldolase	LmxM.16.0760	E9AQN5	36.976	2.221914	2.307883	Carbohydrate transport and metabolism
Paraflagellar rod protein 2C	LmxM.16.1430	E9AQV6	76.114	2.439255	2.304073	Cytoskeleton
Putative electron-transfer-flavoprotein, alpha polypeptide	LmxM.28.1140	E9AZT2	33.555	1.358488	2.289095	Energy production and conversion
Uncharacterized protein	LmxM.02.0450	E9AJK7	29.955	0.478885	2.270905	Function unknown
2,3-bisphosphoglycerate-independent phosphoglycerate mutase	LmxM.36.6650	E9AUA1	60.813	1.25166	2.256762	Carbohydrate transport and metabolism
2,3-diketo-5-methylthio-1-phosphopentane phosphatase, putative	LmxM.36.5910	E9AU26	41.285	1.941203	2.247961	Energy production and conversion
Uncharacterized protein	LmxM.08.1100	E9AMI0	42.21	5.203873	2.242727	Function unknown
Mitochondrial import receptor subunit ATOM69, putative	LmxM.28.2170	E9B038	71.336	2.704644	2.238163	Function unknown
Putative heat-shock protein hsp70	LmxM.28.2770	E9B099	71.24	2.958401	2.233768	Post-translational modification, protein turnover, and chaperones
GTP-binding nuclear protein	LmxM.25.1420	E9AXM1	24.224	1.561282	2.227434	Function unknown
Putative glycosomal membrane protein	LmxM.28.2260	E9B047	24.141	1.229487	2.214022	Function unknown
Putative nucleosome assembly protein	LmxM.19.0440	E9ARZ6	39.825	5.495868	2.213369	Function unknown
Stress-inducible protein STI1 homolog	LmxM.36.0070	E9ASC5	28.977	2.164867	2.209058	Function unknown
Elongation factor-1 gamma	LmxM.09.0970	E9AMU9	46.201	2.064677	2.199931	Post-translational modification, protein turnover, and chaperones
Proteasome regulatory non-ATPase subunit,putative	LmxM.08_29.0120	E9AM67	99.313	4.045086	2.199885	Post-translational modification, protein turnover, and chaperones
Putative RNA binding protein	LmxM.31.0750	E9B2J9	25.249	3.41341	2.194153	Function unknown
Putative cysteinyl-tRNA synthetase	LmxM.12.0250	E9ANV2	88.469	3.424106	2.187315	Translation, ribosomal structure and biogenesis
Putative 60S ribosomal protein L5	LmxM.34.1880	E9B665	34.029	3.180397	2.186111	Translation, ribosomal structure and biogenesis
Putative 14-3-3 protein	LmxM.11.0350	E9ANI1	29.147	1.91562	2.151841	Signal transduction mechanisms
Putative casein kinase	LmxM.34.1010	E9B5Y1	39.933	2.523113	2.149144	Signal transduction mechanisms

DEAD/DEAH box helicase, putative	LmxM.03.0690	E9AJV2	230.86	1.475655	2.138348	Replication, recombination and repair
Elongation initiation factor 2 alpha subunit,putative	LmxM.03.0980	E9AJY2	46.623	2.70084	2.130091	Translation, ribosomal structure and biogenesis
Putative 60S Ribosomal protein L36	LmxM.34.1920	E9B669	11.936	3.002892	2.126666	Translation, ribosomal structure and biogenesis
Succinate--CoA ligase [ADP-forming] subunit alpha, mitochondrial	LmxM.25.2140	E9AXU7	30.944	3.574076	2.122467	Energy production and conversion
Possible lysine decarboxylase, putative	LmxM.15.0040	E9AQ01	37.838	1.301865	2.120824	Function unknown
KH domain containing protein, putative	LmxM.27.1300	E9AZ36	60.007	1.624961	2.120594	Function unknown
Putative 40S ribosomal protein S33	LmxM.26.1630	E9AYE2	9.7461	1.773204	2.101196	Translation, ribosomal structure and biogenesis
Putative GMP synthase (Putative glutamine amidotransferase)	LmxM.22.0110	E9AVJ6	71.256	0.639607	2.100074	Nucleotide transport and metabolism
Putative cystathionine beta-lyase	LmxM.31.2640	E9B343	44.512	3.190256	2.099992	Amino acid transport and metabolism
Putative ribosomal protein L3	LmxM.33.2870	E9B578	47.543	1.526833	2.091579	Translation, ribosomal structure and biogenesis
ATP synthase subunit beta	LmxM.25.1180	E9AXJ7	53.07	4.817381	2.086735	Energy production and conversion
Putative RNA-binding protein	LmxM.34.2200	E9B699	30.286	2.422691	2.075274	Function unknown
Enolase	LmxM.14.1160	E9APW3	46.145	1.882749	2.069273	Carbohydrate transport and metabolism
Putative chaperonin alpha subunit	LmxM.31.3270	E9B3B0	59.189	1.956973	2.068224	Post-translational modification, protein turnover, and chaperones
Elongation factor 2	LmxM.36.0180	E9ASD6	94.056	5.251979	2.067732	Translation, ribosomal structure and biogenesis
Translation initiation factor-like protein	LmxM.08.0550	E9AMD5	38.006	0.818203	2.065882	Translation, ribosomal structure and biogenesis
Trypanothione reductase	LmxM.05.0350	E9AKE1	53.173	2.554178	2.06151	Energy production and conversion
Putative heat shock protein	LmxM.32.2390	E9B478	72.111	2.528226	2.056594	Post-translational modification, protein turnover, and chaperones
Putative small GTP-binding protein Rab1	LmxM.27.0760	E9AYX8	22.254	1.562	2.035763	Function unknown

Receptor-type adenylate cyclase a-like protein	LmxM.36.3180	E9AT96	152.3	1.23933	2.034251	Signal transduction mechanisms
Putative trypanothione synthetase	LmxM.27.1870	E9AZ89	74.397	0.951132	2.021039	Amino acid transport and metabolism
Putative prohibitin	LmxM.16.1610	E9AQX4	30.271	2.865966	2.011059	Post-translational modification, protein turnover, and chaperones
Actin	LmxM.04.1230	E9AKA4	40.483	1.551365	2.007624	Cytoskeleton
Cysteine conjugate beta-lyase,aminotransferase-like protein	LmxM.32.1330	E9B3W9	46.123	1.835807	2.005759	Amino acid transport and metabolism
Putative 60S ribosomal protein L35	LmxM.26.2330	E9AYL4	15.191	1.286664	2.005533	Translation, ribosomal structure and biogenesis
Putative 2-oxoglutarate dehydrogenase subunit	LmxM.27.0880	E9AYZ0	112.78	3.017744	2.004937	Energy production and conversion
40S ribosomal protein S6	LmxM.21.1780	E9AVH4	28.262	3.831138	1.996139	Translation, ribosomal structure and biogenesis
Putative ATP synthase F1 subunit gamma protein	LmxM.21.1770	E9AVH3	34.408	3.292401	1.993364	Energy production and conversion
Uncharacterized protein	LmxM.23.0080	E9AW08	48.444	1.247535	1.98011	Function unknown
Short chain 3-hydroxyacyl-CoA dehydrogenase,putative	LmxM.36.1140	E9ASN4	33.127	1.999506	1.979868	Lipid transport and metabolism
Uncharacterized protein	LmxM.22.0730	E9AVQ7	47.152	1.692706	1.956553	Function unknown
Uncharacterized protein	LmxM.26.1960	E9AYH6	89.474	2.090948	1.955921	Function unknown
Putative ATP synthase	LmxM.36.3100	E9AT88	25.258	0.959622	1.94962	Energy production and conversion
T-complex protein 1 subunit eta	LmxM.34.3860	E9B6R4	61.731	1.59661	1.948791	Post-translational modification, protein turnover, and chaperones
Succinate--CoA ligase [ADP-forming] subunit beta, mitochondrial	LmxM.36.2950	E9AT73	44.097	1.420967	1.944759	Energy production and conversion
Putative heat shock protein	LmxM.18.1370	E9ARS1	91.953	2.99097	1.944446	Post-translational modification, protein turnover, and chaperones
Uncharacterized protein	LmxM.08_29.0320	E9AM47	36.554	2.2243	1.944234	Function unknown
UTP--glucose-1-phosphate uridylyltransferase	LmxM.18.0990	E9ARN6	54.445	1.362505	1.938074	Carbohydrate transport and metabolism

Soluble NSF attachment protein, SNAP, putative	LmxM.02.0310	E9AJJ4	70.815	2.298777	1.936786	Function unknown
Tubulin beta chain	LmxM.32.0792	E9AMJ9	49.723	4.671778	1.935033	Cytoskeleton
Eukaryotic initiation factor 4A-1	LmxM.01.0770	E9AJG4	51.086	2.006898	1.932202	Replication, recombination and repair
Cell division protein kinase 2 homolog CRK1	LmxM.21.1080	E9AV92	34.473	3.728864	1.928258	Signal transduction mechanisms
Malate dehydrogenase	LmxM.29.2490	E9B4I1	33.738	2.393079	1.922505	Energy production and conversion
Putative heat shock 70-related protein 1, mitochondrial	LmxM.29.2490	E9B121	72.521	4.193067	1.915103	Post-translational modification, protein turnover, and chaperones
Uncharacterized protein	LmxM.14.0190	E9APL2	22.315	1.020016	1.914243	Function unknown
Hsp70 protein/TPR repeat, putative	LmxM.08_29.1240	E9ALU9	79.692	1.865965	1.9078	Function unknown
40S ribosomal protein S3a	LmxM.34.0400	E8NHI0	30.036	1.626549	1.904096	Translation, ribosomal structure and biogenesis
Putative fructose-1,6-bisphosphatase, cytosolic	LmxM.04.1160	E9AK97	35.244	1.190344	1.901364	Carbohydrate transport and metabolism
Putative T-complex protein 1, theta subunit	LmxM.36.6910	E9AUC7	58.239	2.064699	1.898678	Post-translational modification, protein turnover, and chaperones
Eukaryotic translation initiation factor 3 subunit a	LmxM.17.0010	E9ARC2	87.627	0.856054	1.898427	Translation, ribosomal structure and biogenesis
40S ribosomal protein S4	LmxM.13.1230	E9APE7	30.654	1.913102	1.897813	Translation, ribosomal structure and biogenesis
Dihydrolipoamide acetyltransferaselike protein	LmxM.21.0550	E9AV22	40.175	3.177068	1.89623	Energy production and conversion
Putative RNA helicase	LmxM.21.1552	E9AVE1	49.469	2.64949	1.895517	Replication, recombination and repair
Activated protein kinase c receptor (LACK),guanine nucleotide-binding protein beta subunit-like protein	LmxM.28.2740	E8NHN2	34.402	1.96924	1.895239	Function unknown
Phenylalanyl-tRNA synthetase alpha chain,putative	LmxM.31.0870	E9B2L1	56.751	2.450794	1.893655	Translation, ribosomal structure and biogenesis
Dihydrolipoyl dehydrogenase	LmxM.31.3310	E9B3B4	50.487	3.212594	1.892649	Energy production and conversion
Uncharacterized protein	LmxM.21.0430	E9AV10	47.492	2.454801	1.889858	Function unknown

Putative proteasome activator protein pa26	LmxM.34.0750	E9B5V5	24.9	0.647269	1.884642	Post-translational modification, protein turnover, and chaperones
Mitochondrial import receptor subunit ATOM46, putative	LmxM.28.0930	E9AZQ9	45.651	1.554141	1.883959	Function unknown
Present in the outer mitochondrial membrane proteome 10	LmxM.09.1010	E9AMV3	67.39	2.092179	1.878873	Function unknown
Putative Gim5A protein (Glycosomal membrane protein)	LmxM.34.3700	E9B6P8	24.88	1.164324	1.867528	Function unknown
Paraflagellar rod component, putative	LmxM.36.4230	E9ATK7	117.74	2.422421	1.863209	Function unknown
Biotin/lipoate protein ligase-like protein	LmxM.30.1070	E9B1Q4	28.493	1.279842	1.853389	Coenzyme transport and metabolism
Aldehyde dehydrogenase, mitochondrial	LmxM.25.1120	E9AXJ1	54.259	3.198088	1.851914	Energy production and conversion
Putative aspartate carbamoyltransferase	LmxM.16.0540	E9AQL2	35.416	0.874609	1.847603	Nucleotide transport and metabolism
Putative nucleolar protein	LmxM.10.0210	E9AN29	52.725	3.16052	1.845518	Translation, ribosomal structure and biogenesis
Nascent polypeptide-associated complex subunit beta	LmxM.36.3770	E9ATF9	11.65	1.643642	1.844212	Transcription
Ribonucleotide reductase M2 subunit	LmxM.27.2050	E9AZB1	40.581	2.478532	1.84263	Nucleotide transport and metabolism
Putative nucleolar RNA helicase II	LmxM.05.0140	E9AKB8	74.299	1.461675	1.833523	Replication, recombination and repair
Uncharacterized protein	LmxM.36.5100	E9ATU4	105.88	1.059517	1.826904	Function unknown
Putative isoleucyl-tRNA synthetase	LmxM.36.5620	E9ATZ6	125.76	2.090341	1.825017	Translation, ribosomal structure and biogenesis
Putative arginyl-tRNA synthetase	LmxM.27.1310	E9AZ37	78.015	2.406632	1.819823	Translation, ribosomal structure and biogenesis
Uncharacterized protein	LmxM.24.1560	E9AX01	67.501	1.44059	1.816796	Function unknown
Succinyl-CoA:3-ketoacid-coenzyme A transferase	LmxM.32.2340	E9B473	52.533	2.892856	1.814759	Lipid transport and metabolism
NADH:flavin oxidoreductase/NADH oxidase,putative	LmxM.12.1130	E9AP13	41.405	3.043852	1.799325	Energy production and conversion

Putative nucleolar RNA binding protein	LmxM.15.1380	E9AQD3	49.101	1.722522	1.787061	Translation, ribosomal structure and biogenesis
T-complex protein 1 subunit gamma	LmxM.23.1220	E9AWE0	60.23	2.627496	1.783028	Post-translational modification, protein turnover, and chaperones
Flagellum targeting protein kharon1	LmxM.36.5850	E9AU20	57.284	1.982078	1.78059	Function unknown
Succinate dehydrogenase [ubiquinone] flavoprotein subunit, mitochondrial	LmxM.24.1630	E9AX08	66.721	0.701838	1.777746	Energy production and conversion
Uncharacterized protein	LmxM.29.2850	E9B159	13.065	1.827792	1.77029	Function unknown
Putative ribosomal protein S20	LmxM.28.1010	E9AZR7	13.011	2.790705	1.769952	Translation, ribosomal structure and biogenesis
Lysine--tRNA ligase	LmxM.15.0230	E9AQ24	67.154	0.764657	1.763756	Translation, ribosomal structure and biogenesis
Putative prolyl-tRNA synthetase	LmxM.18.1210	E9ARQ8	81.231	1.176978	1.761847	Translation, ribosomal structure and biogenesis
Putative asparagine synthetase a (Putative aspartate--ammonia ligase)	LmxM.26.0830	E9AY61	39.829	1.275605	1.753216	Amino acid transport and metabolism
Uncharacterized protein	LmxM.34.5340	E9B760	38.891	0.878808	1.751734	Function unknown
Calpain-like cysteine peptidase, putative	LmxM.14.0850	E8NHG6	79.84	1.866995	1.749101	Post-translational modification, protein turnover, and chaperones
Putative GTP-binding protein	LmxM.08_29.2200	E9ALK3	76.898	1.49938	1.74575	Function unknown
Putative ATP-dependent RNA helicase	LmxM.30.0250	E9B1H5	103.5	0.425856	1.745319	Replication, recombination and repair
Putative 60S ribosomal protein L28	LmxM.11.1130	E8NHE3	16.236	1.057914	1.744705	Translation, ribosomal structure and biogenesis
Putative carbamoyl-phosphate synthase	LmxM.16.0590	E9AQL8	206.02	1.229273	1.737167	Nucleotide transport and metabolism
Putative carboxypeptidase	LmxM.32.2540	E9B493	56.957	1.377572	1.73636	Amino acid transport and metabolism
HEAT repeats, putative	LmxM.18.0700	E9ARK4	77.256	1.064451	1.726466	Signal transduction mechanisms
Putative carboxypeptidase (Metallo-peptidase, clan ma(E), family 32)	LmxM.13.0090	E9AP43	57.25	0.611733	1.72372	Amino acid transport and metabolism

Putative thimet oligopeptidase (Metallo-peptidase, clan ma(E), family m3)	LmxM.26.1570	E9AYD6	77.044	2.246833	1.720401	Amino acid transport and metabolism
mRNA cap guanine-N7 methyltransferase, putative	LmxM.11.0480	E9ANJ4	117.57	0.904531	1.717564	Secondary metabolites biosynthesis, transport, and catabolism
Acetyl-coenzyme A synthetase	LmxM.23.0710	E9AW86	77.449	1.446858	1.704289	Lipid transport and metabolism
Uncharacterized protein	LmxM.31.3010	E9B384	96.957	1.439937	1.704183	Function unknown
Chaperonin HSP60, mitochondrial	LmxM.36.2030	E9ASX8	59.655	3.652107	1.701563	Post-translational modification, protein turnover, and chaperones
Protein phosphatase, putative	LmxM.25.0750	E9AXF3	45.211	0.930229	1.695671	Signal transduction mechanisms
Phosphotransferase	LmxM.21.0250	E9AUZ2	51.67	3.323426	1.670078	Carbohydrate transport and metabolism
Short chain dehydrogenase, putative	LmxM.33.0010	E9B4G7	33.496	2.338987	1.665532	Function unknown
Glutathione peroxidase	LmxM.26.0810	E9AY60	19.257	1.959126	1.659619	Post-translational modification, protein turnover, and chaperones
Putative 60S ribosomal protein L7a	LmxM.07.0500	E9AL62	29.762	1.042543	1.658777	Translation, ribosomal structure and biogenesis
Eukaryotic translation initiation factor 3 subunit l	LmxM.36.0250	E9ASE2	62.709	1.380174	1.658772	Translation, ribosomal structure and biogenesis
Ribonucleoprotein p18, mitochondrial,putative	LmxM.15.0275	E9AQ29	21.244	1.300365	1.65438	Function unknown
Putative 60S ribosomal protein L10a	LmxM.18.0620	E9ARK0	24.578	1.213448	1.653872	Translation, ribosomal structure and biogenesis
Uncharacterized protein	LmxM.33.4010	E9B5J6	12.188	0.57821	1.651432	Function unknown
Dihydrolipoamide acetyltransferase component of pyruvate dehydrogenase complex	LmxM.28.2420	E9B063	41.737	1.374478	1.651415	Energy production and conversion
Isocitrate dehydrogenase [NADP]	LmxM.32.2550	E9B494	46.498	1.155245	1.648111	Energy production and conversion
Phosphoglycerate kinase	LmxM.20.0110	E9AUF1	44.852	1.608891	1.643494	Carbohydrate transport and metabolism
Leucine-rich repeat protein 1, putative	LmxM.28.1990	E9B018	81.564	2.872875	1.642743	Function unknown

Peptidylprolyl isomerase	LmxM.10.0890	E9AN99	16.715	0.838179	1.642567	Post-translational modification, protein turnover, and chaperones
Putative 40S ribosomal protein S17	LmxM.28.2555	E9B078	16.408	2.833026	1.637931	Translation, ribosomal structure and biogenesis
Putative ATPase alpha subunit	LmxM.05.0500	E8NHQ7	62.599	2.988506	1.630674	Energy production and conversion
Proteasome subunit alpha type	LmxM.34.4850	E9B713	27.265	0.793793	1.62944	Post-translational modification, protein turnover, and chaperones
Putative 40S ribosomal protein S9	LmxM.36.1250	E9ASP5	22.099	6.643364	1.621068	Translation, ribosomal structure and biogenesis
Putative cystathione gamma lyase	LmxM.34.3230	E9B6K2	44.446	1.567654	1.618456	Amino acid transport and metabolism
Putative vacuolar-type proton translocating pyrophosphatase 1	LmxM.30.1220	E9B1S0	83.722	2.974345	1.607136	Energy production and conversion
Flagellar Member 8	LmxM.32.3070	E9B4E7	96.46	0.408057	1.604774	Function unknown
40S ribosomal protein S19-like protein	LmxM.08_29.2860	E9ALD8	18.153	1.092507	1.592513	Translation, ribosomal structure and biogenesis
Regulatory subunit of protein kinase a-like protein	LmxM.33.2820	E9B573	71.735	4.010987	1.589987	Signal transduction mechanisms
Uncharacterized protein	LmxM.31.0950	E9B2L9	102.25	1.072074	1.588059	Replication, recombination and repair
40S ribosomal protein S12	LmxM.13.0570	E9AP81	15.592	2.731656	1.583693	Translation, ribosomal structure and biogenesis
Chaperonin HSP60, mitochondrial	LmxM.36.2020	E9ASX7	60.305	0.803869	1.57259	Post-translational modification, protein turnover, and chaperones
Chaperonin containing t-complex protein,putative	LmxM.31.1000	E9B2M5	59.3	1.346652	1.552247	Post-translational modification, protein turnover, and chaperones
Putative orotidine-5-phosphate decarboxylase/orotate phosphoribosyltransferase (Putative ompdcase-oprtase)	LmxM.16.0550	E9AQL3	49.635	1.430818	1.552148	Nucleotide transport and metabolism
Putative glycyl tRNA synthetase	LmxM.36.3840	E9ATG6	70.262	0.802381	1.548953	Translation, ribosomal structure and biogenesis
Ankyrin repeats (3 copies), putative	LmxM.08_29.1100	E9ALW3	40.08	0.747105	1.547458	Function unknown

Calpain-like cysteine peptidase	LmxM.20.1180	E9AUQ7	103.53	0.763158	1.543974	Post-translational modification, protein turnover, and chaperones
Fructose-bisphosphate aldolase	LmxM.36.1260	E9ASP6	40.833	1.737608	1.540751	Carbohydrate transport and metabolism
Putative dynein heavy chain	LmxM.13.1650	E9API9	530.22	0.743998	1.536112	Cytoskeleton
Uncharacterized protein	LmxM.36.3620	E9ATE1	11.807	0.590263	1.532432	Function unknown
Putative peptidyl dipeptidase	LmxM.02.0740	E9AJN6	76.626	2.837368	1.522149	Amino acid transport and metabolism
Eukaryotic translation initiation factor 4 gamma 5	LmxM.10.1080	E9ANB8	88.651	0.589253	1.517088	Translation, ribosomal structure and biogenesis
Uncharacterized protein	LmxM.33.1520	E9B4X7	14.324	2.614089	1.514779	Function unknown
3-hydroxy-3-methylglutaryl coenzyme A reductase	LmxM.29.3190	E9B193	45.766	3.388206	1.497988	Lipid transport and metabolism
40S ribosomal protein S8	LmxM.24.2070	E9AX53	24.686	0.458656	1.493217	Translation, ribosomal structure and biogenesis
Putative cysteine synthase	LmxM.36.3590	E9ATD8	35.418	0.969154	1.485382	Amino acid transport and metabolism
Proteasome regulatory non-ATP-ase subunit 2,putative	LmxM.28.1730	E9AZZ2	107.86	2.069311	1.481483	Post-translational modification, protein turnover, and chaperones
40S ribosomal protein SA	LmxM.36.5010	E9ATT5	27.569	3.602248	1.474768	Translation, ribosomal structure and biogenesis
Putative small GTP-binding protein	LmxM.05.0030	E9AKA7	21.716	1.091374	1.467255	Intracellular trafficking, secretion, and vesicular transport
Putative seryl-tRNA synthetase	LmxM.11.0100	E9ANF6	52.907	2.284783	1.459522	Translation, ribosomal structure and biogenesis
Putative ATP-dependent DEAD/H RNA helicase	LmxM.22.1500	E9AVY2	120.17	1.691	1.459062	Replication, recombination and repair
Putative asparaginyl-tRNA synthetase	LmxM.33.2340	E9B520	98.674	3.479636	1.456731	Translation, ribosomal structure and biogenesis
Putative chaperonin TCP20	LmxM.13.1660	E9APJ0	58.997	2.053589	1.451502	Post-translational modification, protein turnover, and chaperones
Proteasome regulatory non-ATP-ase subunit,putative	LmxM.19.1120	E9AS63	46.506	2.577909	1.45057	Post-translational modification, protein turnover, and chaperones
Polyadenylate-binding protein	LmxM.34.5040	E9B730	62.989	1.559968	1.449484	Function unknown

Citrate synthase	LmxM.18.0670	E9ARK7	50.193	0.704938	1.446531	Energy production and conversion
Transitional endoplasmic reticulum ATPase,putative	LmxM.36.1370	E9ASQ6	86.887	1.172815	1.443235	Post-translational modification, protein turnover, and chaperones
Adenosylhomocysteinase	LmxM.36.3910	E9ATH3	47.871	1.841103	1.437763	Coenzyme transport and metabolism
Cytochrome c oxidase subunit IV	LmxM.12.0670	E9ANZ4	39.012	0.923635	1.430027	Post-translational modification, protein turnover, and chaperones
60S ribosomal protein L18a	LmxM.34.0600	E9B5U1	20.779	2.161256	1.426924	Translation, ribosomal structure and biogenesis
Putative vacuolar ATP synthase subunit b	LmxM.28.2430	E9B064	55.529	0.991172	1.415684	Energy production and conversion
Putative 40S ribosomal protein S11	LmxM.21.1550	E9AUU6	16.326	1.514666	1.409504	Translation, ribosomal structure and biogenesis
Putative ATP-dependent RNA helicase	LmxM.34.3100	E9B6I9	100.88	2.223118	1.401527	Replication, recombination and repair
Putative 3-ketoacyl-CoA thiolase-like protein	LmxM.30.1630	E9B1W3	42.996	0.454528	1.39539	Lipid transport and metabolism
Putative aminopeptidase P	LmxM.34.2350	E9B6B5	53.78	0.413423	1.39101	Amino acid transport and metabolism
Putative ATP-dependent RNA helicase	LmxM.31.0400	E9B2G1	67.786	2.908208	1.390901	Replication, recombination and repair
Uncharacterized protein LMXM_28_3030	LmxM.28.3030	E9B0C5	147.33	1.574886	1.3876	Function unknown
Putative immunodominant antigen (Tc40 antigen-like)	LmxM.14.0930	E9APT8	91.393	0.682644	1.382318	Function unknown
High mobility group protein homolog tdp-1,putative	LmxM.08_29.0850	E9ALZ2	33.7	0.578143	1.375416	Chromatin structure and dynamics
Nucleoside diphosphate kinase	LmxM.31.2950	E9B376	16.674	2.1986	1.367537	Nucleotide transport and metabolism
Putative threonyl-tRNA synthetase	LmxM.34.1410	E9B620	89.729	0.627649	1.355424	Translation, ribosomal structure and biogenesis
Uncharacterized protein	LmxM.09.0220	E9AMM1	93.386	0.993703	1.351886	Inorganic ion transport and metabolism
Ubiquitin-conjugating enzyme-like protein	LmxM.13.1580	E9API2	16.033	1.180286	1.341337	Post-translational modification, protein turnover, and chaperones
Putative aminopeptidase	LmxM.19.0160	E9ARW7	42.47	1.817831	1.327777	Translation, ribosomal structure and biogenesis

S-adenosylmethionine synthase	LmxM.29.3500	E9B1C6	43.128	2.559999	1.323151	Coenzyme transport and metabolism
GrpE protein homolog	LmxM.29.0730	E9B0J8	23.867	0.432971	1.322921	Post-translational modification, protein turnover, and chaperones
Putative histone H3 variant	LmxM.19.0630	E9AS15	16.554	1.231835	1.317073	Chromatin structure and dynamics
Elongation of fatty acids protein	LmxM.14.0670	E9APR2	32.819	0.403084	1.312091	Lipid transport and metabolism
Uncharacterized protein	LmxM.36.4910	E9ATS5	43.007	0.520591	1.302684	Function unknown
Peptidyl-prolyl cis-trans isomerase	LmxM.25.0910	E9AXG9	18.794	2.065751	1.301119	Post-translational modification, protein turnover, and chaperones
Proteasome subunit beta	LmxM.06.0140	E9AKP2	27.909	1.184801	1.30003	Post-translational modification, protein turnover, and chaperones
Uncharacterized protein	LmxM.32.2440	E9B483	56.275	0.779304	1.295357	Function unknown
6-phosphogluconate dehydrogenase, decarboxylating	LmxM.34.3340	E9B6L3	52.168	2.540756	1.295049	Carbohydrate transport and metabolism
Heat shock protein 70-related protein	LmxM.26.1240	E9AYA3	70.599	1.789123	1.290277	Post-translational modification, protein turnover, and chaperones
Putative T-complex protein 1, beta subunit	LmxM.27.1260	E9AZ29	57.734	1.150429	1.29011	Post-translational modification, protein turnover, and chaperones
Glycosomal phosphoenolpyruvate carboxykinase,putative	LmxM.27.1810	E9AZ83	58.379	1.466229	1.282989	Energy production and conversion
Transketolase	LmxM.24.2060	E9AX52	71.827	1.150941	1.279756	Carbohydrate transport and metabolism
Plasma membrane ATPase	LmxM.18.1520	E9ART6	107.51	2.18918	1.26917	Inorganic ion transport and metabolism
Putative mitochondrial phosphate transporter	LmxM.18.1520	E9B6X2	34.712	1.146334	1.265716	Function unknown
Mitochondrial processing peptidase, beta subunit,putative,metallo-peptidase, Clan ME, Family M16	LmxM.34.1380	E9B617	54.641	3.741147	1.258868	Post-translational modification, protein turnover, and chaperones
Cytoskeleton associated protein, putative	LmxM.14.1440	E9APZ2	108.73	3.527443	1.244072	Transcription
Ribosomal protein L37	LmxM.32.1955	E9B433	9.8354	0.898885	1.238888	Translation, ribosomal structure and biogenesis

Glyceraldehyde-3-phosphate dehydrogenase	LmxM.29.2980	E9B170	39.123	1.678564	1.238664	Carbohydrate transport and metabolism
Uncharacterized protein	LmxM.02.0550	E9AJL7	17.561	1.168122	1.23853	Function unknown
Uncharacterized protein	LmxM.25.2410	E9AXX5	109.59	3.009351	1.237481	Function unknown
Putative mitochondrial intermediate peptidase	LmxM.36.4450	E9ATM9	75.893	1.01479	1.234832	Amino acid transport and metabolism
Putative eukaryotic translation initiation factor 3 subunit 8	LmxM.36.6980	E9AUD5	81.701	0.652416	1.234017	Translation, ribosomal structure and biogenesis
Isocitrate dehydrogenase [NADP]	LmxM.10.0290	E9AN39	48.403	0.79508	1.230407	Energy production and conversion
40S ribosomal protein S2	LmxM.31.0450	E9B2G6	28.634	5.266041	1.218673	Translation, ribosomal structure and biogenesis
Putative long-chain-fatty-acid-CoA ligase	LmxM.01.0520	E9AJD9	77.583	1.487818	1.218114	Lipid transport and metabolism
Putative 60S ribosomal protein L27A/L29	LmxM.34.3760	E9B6Q4	16.07	2.082184	1.199398	Translation, ribosomal structure and biogenesis
Putative 60S ribosomal protein L17	LmxM.24.0040	E9AWJ4	19.083	1.252964	1.198661	Translation, ribosomal structure and biogenesis
Histone H2B	LmxM.19.0030	E9ARV5	11.921	2.703139	1.193329	Chromatin structure and dynamics
Oligopeptidase b	LmxM.09.0770	E9AMS8	83.587	2.482506	1.192837	Amino acid transport and metabolism
Putative carnitine/choline acetyltransferase	LmxM.08_29.1310	E9ALU2	68.576	1.228351	1.19008	Function unknown
Putative valyl-tRNA synthetase	LmxM.29.3130	E9B187	109.79	1.067616	1.176959	Translation, ribosomal structure and biogenesis
Heat shock protein 83-1	LmxM.32.0312	E9B3L2	80.572	1.842091	1.173089	Post-translational modification, protein turnover, and chaperones
Tubulin alpha chain	LmxM.13.0280	E9AP62	60.184	3.248999	1.172007	Cytoskeleton
Mitochondrial processing peptidase alpha subunit,putative	LmxM.32.2610	E9B4A1	53.211	2.114993	1.152941	Post-translational modification, protein turnover, and chaperones
Myo-inositol-1-phosphate synthase	LmxM.14.1360	E9APY4	58.19	0.61717	1.149902	Lipid transport and metabolism
Putative aminopeptidase (Metallo-peptidase, clan mf, family m17)	LmxM.11.0630	E9ANK8	56.931	0.657044	1.149468	Amino acid transport and metabolism
Glutamine synthetase	LmxM.06.0370	E9AKR5	42.292	0.935855	1.13762	Amino acid transport and metabolism

Ubiquitin-fusion protein	LmxM.30.1900	E9B1Y9	14.682	1.694145	1.136152	Post-translational modification, protein turnover, and chaperones
CCR4-NOT transcription complex subunit 1	LmxM.21.0800	E9AV48	249.23	0.766824	1.134027	Transcription
Mkiaa0324 protein-like protein	LmxM.36.5820	E9AU16	56.471	0.915201	1.122429	Function unknown
Protein disulfide-isomerase	LmxM.36.6940	E9AUD1	52.188	1.359149	1.120491	Post-translational modification, protein turnover, and chaperones
Histone H2A	LmxM.08_29.1740	E9ALP9	13.96	1.795312	1.106219	Chromatin structure and dynamics
Putative eukaryotic release factor 3	LmxM.11.1170	E9ANQ6	84.727	1.043996	1.104085	Translation, ribosomal structure and biogenesis
Uncharacterized protein	LmxM.24.2110	E9AX57	55.329	1.125663	1.096315	Lipid transport and metabolism
60S acidic ribosomal protein P0	LmxM.27.1380	E8NHJ8	34.636	1.264811	1.09217	Translation, ribosomal structure and biogenesis
Centromere/microtubule binding protein cbf5,putative	LmxM.21.1760	E9AVH2	48.443	1.61847	1.083178	Translation, ribosomal structure and biogenesis
NAD-specific glutamate dehydrogenase	LmxM.15.1010	E9AQA0	114.65	1.960042	1.075937	Amino acid transport and metabolism
Paraflagellar rod protein 1D	LmxM.08_29.1750	E9ALP8	68.976	1.587347	1.074741	Function unknown
Stress-induced protein sti1	LmxM.08.1110	E9AMI1	62.095	0.694277	1.067927	Function unknown
Putative adenosine kinase	LmxM.29.0880	E9B0L7	37.219	0.920497	1.066509	Carbohydrate transport and metabolism
Methyltransferase	LmxM.36.2390	E9AT16	39.825	0.669715	1.062605	Function unknown
Clathrin heavy chain	LmxM.36.1630	E9AST4	191.23	1.007508	1.060031	Intracellular trafficking, secretion, and vesicular transport
Histone H3	LmxM.10.0870	E9AN97	14.686	1.582322	1.047947	Chromatin structure and dynamics
2-oxoisovalerate dehydrogenase subunit alpha	LmxM.21.1430	E9AVC7	53.225	0.717075	1.045809	Energy production and conversion
Putative aldehyde dehydrogenase	LmxM.29.2900	E9B163	63.914	0.740136	1.043495	Energy production and conversion
Putative vesicle-fusing ATPase	LmxM.20.0810	E9AUL9	81.16	0.72283	1.010024	Post-translational modification, protein turnover, and chaperones
Uncharacterized protein	LmxM.31.2150	E9B2Z5	117.52	1.036656	1.002541	Intracellular trafficking, secretion, and vesicular transport

Starvation-responsive NSPs identified in *Leishmania mexicana* promastigotes following 2 hour duration of nutrient deprivation. The NSPs are listed in the descending order of the fold change in abundance (log2 scale) relative to protein expression in non-starved parasites.

[a]Gene ID according to the GeneDB: The Sanger Institute Pathogen Genomics Database (www.genedb.org).

[b]Protein ID according to the Universal Protein Resource (UniProt) (www.uniprot.org).

[c]Functional classification determined by eggNOG database.
